# Optical genome mapping improves detection and characterisation of cytogenetic abnormalities in non‐Hodgkin lymphomas

**DOI:** 10.1111/bjh.70575

**Published:** 2026-05-24

**Authors:** Coura Fall, Agnès Daudignon, Séverine Valmary‐Degano, Julie Mondet, Lucile Bussot, Lysiane Molina, Simon Chevalier, Edouard Bonneville, Pierre Aubert, Sylvie Tondeur, Clémentine Legrand, Jean‐Baptiste Gaillard, Hélène Guermouche, Anouk Emadali, Sylvain Carras, Christine Lefebvre

**Affiliations:** ^1^ Department of Biological Hematology, Hematological Genetics Unit Grenoble Alpes University Hospital Grenoble France; ^2^ Institute of Medical Genetics Lille University Hospital Lille France; ^3^ Hematology and Immunology Laboratory Valenciennes University Hospital Valenciennes France; ^4^ Department of Pathology Grenoble Alpes University Hospital Grenoble France; ^5^ INSERM U1209, CNRS UMR 5309, Institute for Advanced Biosciences (IAB) University Grenoble Alpes Grenoble France; ^6^ Department of Clinical Hematology Grenoble‐Alpes University Hospital Grenoble France; ^7^ Department of Biological Hematology, Cell Hematology Unit Grenoble Alpes University Hospital Grenoble France; ^8^ Department of Immunology, Flow Cytometry Immunophenotyping Unit Grenoble Alpes University Hospital Grenoble France; ^9^ Department of Molecular Genetics and Cytogenomics, Hematological Genetics Unit Montpellier University Hospital Montpellier France; ^10^ Health Research and Innovation Grenoble Alpes University Hospital Grenoble France

**Keywords:** cytogenetics, FISH, karyotype, non‐Hodgkin lymphoma, optical genome mapping

## Abstract

Classical cytogenetics (CC) encompassing fluorescence in situ hybridisation (FISH) and karyotype remains central to the diagnostic work‐up of non‐Hodgkin lymphoma (NHL). However, karyotype is limited by restricted access to fresh tumour tissue and low resolution, while FISH provides targeted information. Optical genome mapping (OGM) has emerged as a genome‐wide, high‐resolution approach capable of detecting both translocations and copy number variations in a single assay. To assess its feasibility and diagnostic performance in routine practice, we prospectively compared OGM to CC in 105 patients with NHL. OGM was technically feasible in both fresh and frozen tissue specimens. Among 511 cytogenetic abnormalities (CAs) directly compared, 91.1% were concordant between the two methods. Using the low allele fraction guided assembly pipeline, OGM identified all 77 immunoglobulin loci rearrangements, with a sensitivity threshold of 5%. Overall, OGM detected diagnostically relevant CAs in 99/105 patients (94.2%). OGM outperformed CC in 10/105 patients (9.5%), revealing cryptic rearrangements involving major target genes (*MYC*, *BCL2* and *BCL6*) as well as diagnostically relevant CAs in cases with uncertain diagnoses or non‐informative karyotypes. In conclusion, OGM demonstrated feasibility in routine practice and superior diagnostic performance compared with CC, supporting its integration into the standard genetic work‐up of NHL.

## Graphical abstract

Optical genome mapping (OGM) is feasible on fresh and frozen tissue lymphoma samples and demonstrated its added value for accurate diagnostic classification. OGM surpasses karyotype and FISH in refining diagnoses of lymphoma, identifying class‐defining rearrangements along with prognostic markers.

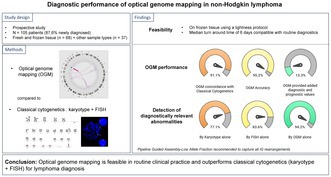

## INTRODUCTION

Accurate diagnosis of non‐Hodgkin lymphomas (NHLs) relies on the integrated assessment of histopathology, cytomorphology, immunophenotyping, cytogenetics and molecular data, together with clinical features. The fifth edition of the World Health Organization classification of haematolymphoid tumours (WHO‐HAEM5) and the International Consensus Classification (ICC) both introduce new or provisional cytogenetic entities, such as large B‐cell lymphoma with *IRF4* rearrangement (LBCL‐IRF4) and high‐grade B‐cell lymphoma with 11q aberration (HGBL‐11q), underscoring the central role of cytogenetic abnormalities (CAs) and molecular alterations in defining NHL subtypes.^1^, ^2^

Accordingly, haematopathologists increasingly rely on fluorescence in situ hybridisation (FISH), karyotyping and chromosomal microarray analysis (CMA), together with next‐generation sequencing (NGS) to inform diagnostic and therapeutic decisions. Detection of rearrangements involving *MYC*, *BCL2*, *BCL6*, *IRF4* as well as 11q aberration is essential for the classification of B‐cell lymphomas (B‐NHL) with high‐grade or large B‐cell morphology.^1^, ^2^, ^3^, ^4^, ^5^ These abnormalities may also predict disease aggressiveness and influence treatment strategies, as illustrated by high‐grade B‐cell lymphoma with *MYC*‐r (‐r, rearrangement) and *BCL2*‐r and/or *BCL6*‐r (HGBL‐MYC/BCL2), which is associated with poor outcome.^1^, ^2^, ^6^ In addition, complex karyotype (CK) is an adverse prognostic factor in mantle cell lymphoma (MCL), lymphoplasmacytic lymphoma and chronic lymphocytic leukaemia (CLL).^7^, ^8^, ^9^, ^10^, ^11^

Optical genome mapping (OGM) has recently emerged as a high‐resolution technique capable of detecting copy number variations (CNVs) and structural variants (SVs), including cryptic events and partner genes of enhancer‐driven rearrangements, thereby challenging the traditional paradigm of targeted FISH testing.^12^, ^13^, ^14^, ^15^, ^16^, ^17^, ^18^

OGM also overcomes key limitations of karyotyping, notably poor chromosomal resolution (5–10 Mb) and some non‐informative results. Although karyotyping can be readily performed on bone marrow aspiration (BM) or peripheral blood (PB) samples and complemented by metaphase FISH to resolve cryptic insertions, its application to tissue‐based lymphomas is limited by restricted access to fresh, unfixed material.^12^, ^13^, ^14^, ^15^, ^16^, ^17^ CMA, conversely, does not detect balanced abnormalities, limiting its diagnostic utility in NHL.^19^, ^20^

Several studies have reported strong concordance between OGM and standard cytogenetic approaches across haematological malignancies (HM), including NHL in a recent series.^21^, ^22^, ^23^, ^24^, ^25^, ^26^, ^27^, ^28^ However, the clinical impact of OGM on diagnostic refinement and prognostic assessment in NHL has not yet been prospectively evaluated in comparison with classical cytogenetics (CC).

In this study, we leveraged a multidisciplinary diagnostic network enabling systematic fresh tissue processing for CC, including karyotype and FISH. Using diverse sample types, we conducted a large prospective study to assess both the feasibility of integrating OGM into clinical practice and to evaluate its diagnostic performance and clinical utility.

## MATERIALS AND METHODS

### Patients and samples

This prospective study included 105 consecutive adult patients diagnosed with NHL at Grenoble Alpes University Hospital between April 2023 and December 2024. The principles of the Declaration of Helsinki were followed, and the study complied with the French General Data Protection Regulation. Informed consent was obtained from all patients in accordance with national (French National Data Protection Commission, CNIL, number 2212382) and local (Grenoble Alpes University Hospital, number 2205066) approvals.

Inclusion criteria were a confirmed diagnosis of NHL, sufficient material for complete diagnostic testing and OGM, with >50% cell viability for fresh tissue samples. Samples comprised tissue biopsies (*n* = 68), BM aspirates (*n* = 7), PB (*n* = 29) and one ascitic fluid (F) sample. Specimens were collected at diagnosis, relapse or histological transformation (Table 1). Whenever possible, tissue samples were prioritised. All samples were processed in parallel for CC and OGM.

**TABLE 1:** Characteristics of the study patients.

No. of patients	105
Age, median years (range)	68.5 (24–90)
Sex ratio (M/F)	1.6 (64/41)
Status	
Diagnosis	92
Relapse	10
Transformation	3
Origin of sample	
Tissue	68
Peripheral blood	29
Bone marrow aspiration	7
Ascites fluid	1
Tumour cell content, median % (range)	60 (3–90)
Lymphoma subtype	
Follicular lymphoma	23
Grade 1/2	15
Grade 3A	8
Mantle cell lymphoma (MCL)	18
Classical MCL	8
Pleomorphic MCL	2
Blastoid MCL	2
Non‐nodal MCL	6
Diffuse large B‐cell lymphoma	18
GCB subtype	8
Non‐GCB subtype	9
NOS, EBV+	1
Marginal zone lymphoma (MZL)	14
Splenic MZL	11
Nodal MZL	2
Extra‐nodal MZL	1
Chronic lymphocytic leukaemia	10
Burkitt lymphoma	5
Transformation of indolent lymphoma	3
Other B‐cell lymphoproliferative disorders	2
Lymphoplasmacytic lymphoma	2
High‐grade B‐cell lymphoma, NOS	2
Plasmablastic lymphoma	2
Large B‐Cell lymphoma with *IRF4* rearrangement	2
T‐cell lymphoma	4

Abbreviations: EBV, Epstein Barr Virus; GCB, Germinal Centre B‐cell, according to the Hans algorithm; NOS, not otherwise specified.

### Diagnosis and classical cytogenetics

All cases underwent comprehensive diagnostic evaluation, including clinical assessment, haematopathology, immunohistochemistry, cytomorphology, flow cytometry, cytogenetics and molecular studies. Tissue lymphomas were reviewed within the French *Lymphopath* network. Diagnoses were established according to the WHO‐HAEM5 criteria following multidisciplinary review.^1^ In addition, ICC criteria were applied for grading follicular lymphoma (FL).^2^ Tumour cell content was assessed by flow cytometry.

**FIGURE 1:**
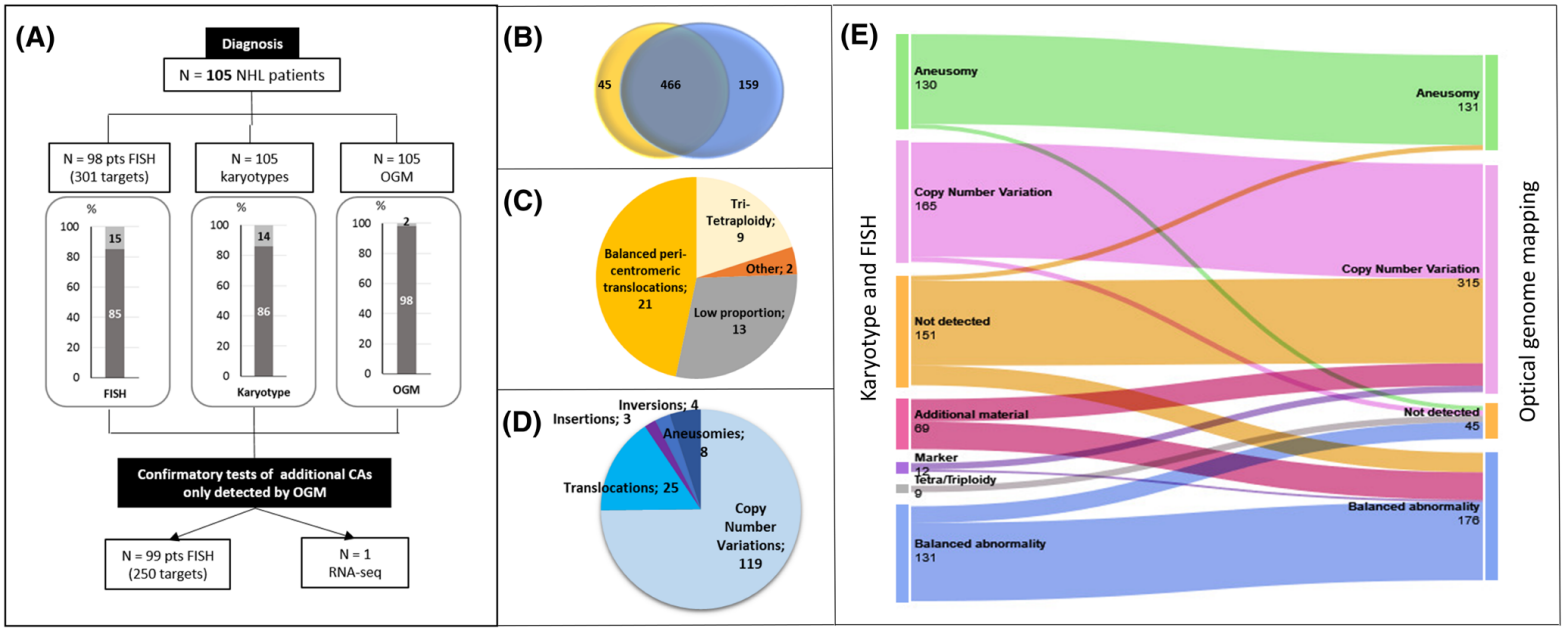
Cytogenetic investigations and comparison with OGM data in the NHL cohort. (A) Flowchart of the cohort showing the initial diagnostic investigations (karyotype, FISH and OGM), including their respective rates of informative results among the 105 NHL patients: 85% for FISH, 86% for karyotype and 98% for OGM (shown in dark grey). Subsequent orthogonal analyses (FISH and RNA (RiboNucleic Acid) sequencing) were performed to investigate CAs only detected by OGM. (B) Venn diagram illustrating CAs initially detected by both OGM and classical cytogenetics (CC) (*n* = 466), CAs not detected by OGM (*n* = 45), and novel CAs identified only by OGM (*n* = 159), subsequently confirmed by FISH. (C) Nature and number of the 45 CAs not detected by OGM. (D) Nature and number of the 159 novel CAs identified by OGM. (E) Flow diagram showing concordance of CAs based on their type and number, and classification of CAs by OGM including abnormalities not detected or not specified by CC. CAs, chromosomal abnormalities; NHL, Non‐Hodgkin Lymphoma; OGM, optical genome mapping; pts, patients; FISH, Fluorescence In Situ Hybridisation. [Colour figure can be viewed at wileyonlinelibrary.com]

R‐banded karyotyping was performed for all patients. Diagnostic FISH analyses (*n* = 301) were carried out in 98 patients, including IGH/MYC and IGH/BCL2 dual‐fusion probes to identify cryptic rearrangements.^12^, ^13^ Orthogonal FISH tests were undertaken to confirm OGM findings (Figure 1A; Table S1). Cytogenetic results were reported according to the International System for Human Cytogenomic Nomenclature (ISCN 2020).^29^

### Optical genome mapping

OGM was performed for all patients. Among the 68 tissue samples, 10 were analysed from frozen material. Ultra‐high‐molecular‐weight (UHMW) genomic DNA (Deoxyribonucleic Acid) was manually extracted from 5 to 8 mg of frozen tissue or 1.5–2.10^6^ cells. DNA was labelled, counterstained and loaded onto Bionano G3.3 chips. DNA scanning on a Saphyr instrument achieved ~300X effective genome coverage, allowing detection of CAs at a 5% allele frequency.

Both the rare variant analysis (RVA) and low allele frequency guided assembly (LAF‐GA) pipelines were applied. Results were interpreted with Bionano Access (version 1.8.1) using the GRCh38 reference genome. We further filtered all confident CAs and applied a custom‐made browser extensible data (BED) file that contains regions and genes recurrently altered in B‐NHL (Figure S1, Table S2). Genomic complexity by OGM was defined when ≥10 CAs (>250 kb) were detected. Results were reported according to the ISCN 2024.^30^ Further details are provided in the Supplemental Material.

### Statistics

Continuous variables were tested for statistical significance using Mann–Whitney tests. Categorical data were compared by chi‐square or Fisher's exact tests. All analyses were performed using GraphPad Prism.

## RESULTS

### Patient characteristics and cytogenetic data

Patient, disease and sample characteristics are summarised in Table 1. NHL subtype distribution reflected the real clinical setting, with slightly fewer diffuse large B‐cell lymphoma (DLBCL, *n* = 18) and a high proportion of FL grade 3A (*n* = 8). Median tumour infiltration was 60% (range 4–90).

A total of 706 CAs were identified by CC: 659 by karyotype and 47 by diagnostic FISH. Seventy diagnostically relevant rearrangements were detected, including t(14;18)/IGH*::BCL2*, t(11;14)/IGH*::CCND1*, v*::BCL6*‐r, IG*::MYC*‐r (*n* ≥ 10, each) and t(6;14)/IGH*::IRF4* (*n* = 2). IGH partner genes remained unresolved in seven cases. Frequent CNVs (*n* ≥ 10) included gains of *BCL2/*18q21, *BCL6/*3q27, *MYC/*8q24, trisomy 12 and losses of 6q, *TP53*/17p13, *DLEU1‐*2/13q14 and *CDKN2A*/9p21.

Overall, karyotype and diagnostic FISH were informative in 90/105 (86%) and 80/98 (85%) patients respectively (Figure 1A).

### OGM is technically feasible in routine practice

A simplified in‐house protocol enabled was used for frozen tissue, reducing cell preparation time and enabling multi‐specimen batching (Supplemental Material).

Median OGM processing time was 6.3 days (range 5–11). Turnaround times (TAT) were comparable across sample types (BM/PB/F vs. fresh tissue vs. frozen tissue) (Figure S2A). CC and OGM showed similar median TAT (13 days vs. 15 days, respectively).

Quality metrics met expected standards; N50 (DNA length ≥150 kb) values were similar across sample types, although coverage and map rates were slightly higher for BM/PB/F samples (Figure S2B–D).

### CC and OGM results are concordant

Across the cohort, OGM was concordant with CC for 103/105 patients (98.1%). OGM initially called a total of 7613 CAs. After filtering and exclusion of SVs arising from complex genomes, 511 CAs were directly comparable, of which 466 (91.1%) were concordant (Figure 1B; Figure S1).

Forty‐five discordant CAs (8.8%) were not detected by OGM, mainly low‐level clones (Figure 1C). Two patients had subclonal *MYC*‐r (3% and 4%) detected by diagnostic FISH but not by OGM or FFPE (Formalin‐fixed, paraffin‐embedded) FISH; patient #32 carried a tetraploid karyotype with IGH::*MYC*‐r present in one copy, resulting in an allelic fraction <2%. OGM also missed subclonal *TP53* deletions in two patients (6% and 8%, by FISH). Two FL cases had focal *TNFRSF14* deletions undetected by OGM owing to polymorphic labelling at the 1p36.33 region (Figure S3).

For critical rearrangements (*BCL2, BCL6, MYC, CCND1* and *IRF4*; 59 samples), OGM was fully concordant with CC.

### OGM detects additional CAs and all IG translocations

Beyond concordant findings, OGM detected additional abnormalities in 89/105 cases (85%). Regarding performance values, sensitivity, specificity and accuracy of OGM were 94%, 100% and 95.2% respectively.

**FIGURE 2:**
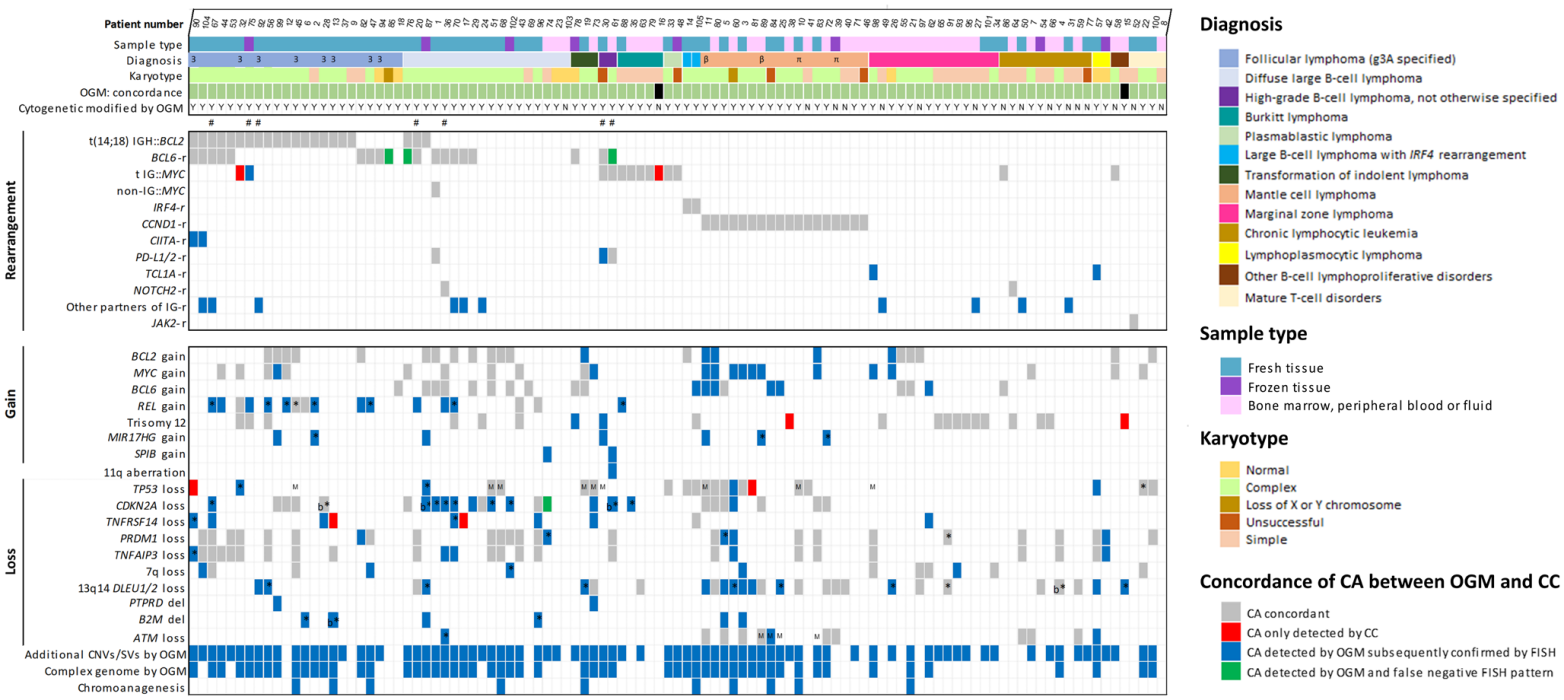
Landscape of the main structural variants, copy number variations and aneuploidies identified by classical cytogenetics and/or OGM in NHL. Heat map depicting the distribution of recurrent CAs detected by CC and OGM. Each column represents an individual NHL patient. Patients are ordered according to final diagnosis. The upper panel show characteristics of patients (number, sample type, diagnosis), karyotype classification (complex, simple, normal, unsuccessful, isolated loss of X or Y chromosome), concordance of OGM with CC (pale green) or discordance (black) and any modification of the cytogenetic formula (Y, yes; N, no). OGM concordance means that the most diagnostically relevant CAs were detected by both OGM and CC. The middle and lower panels display recurrently altered loci or chromosomal regions. The middle panel shows recurrent gains, and the lower panel shows losses. Only the most frequent altered loci and regions are listed. A colour code indicates concordance or discordance of CAs between CC and OGM. Of note, the two *TP53* deletion not detected by OGM corresponded to low‐level clones. Ten cases of chromoanagenesis were identified by OGM. Asterisks indicate a submicroscopic CNV <10 Mb (gain or loss); b, biallelic loss; for follicular lymphomas, 3 denotes grade 3A; β indicates blastoid mantle cell lymphoma; π indicates pleomorphic mantle cell lymphoma; # indicates a dual IG rearrangement in a single case; M indicates *TP53* or *ATM* variant detected by targeted NGS; CA, chromosomal abnormality; CC, classical cytogenetics (karyotype + FISH); OGM, optical genome mapping; FISH, fluorescence in situ hybridisation. [Colour figure can be viewed at wileyonlinelibrary.com]

Among the 228 new CAs detected by OGM, 159 were assessable and confirmed by FISH (Figure 1D), except for one small (74 kb) *CDKN2A* deletion (false negative by FISH). Overall, OGM clarified unresolved abnormalities by identifying markers, rings or unknown chromosomal material (Figure 1E). OGM identified 118 additional CNVs: 67 large (≥10 Mb) and 51 submicroscopic (<10 Mb). Frequent large CNVs included gains of 18q/*BCL2*, 3q/*BCL6* and 8q/*MYC*. Recurrent submicroscopic CNVs included deletions of *DLEU1‐2*/13q14, *CDKN2A*/9p21.3, *B2M*/15q21, *TP53*/17p13 and gains of *REL*/2p16 and *MIR17HG*/13q31 (Figure 2).

OGM identified all partner genes rearranged with *BCL2*, *BCL6*, *MYC* and IG (immunoglobulin) loci (Figure 2; Table S3). OGM revealed one additional IGH*::MYC*‐r in an FL (#75) and an 11q aberration in an elderly patient with HGBL‐MYC/BCL6 (#61) as well as rare and novel IG‐r involving *FCGR2B*, *REL*, *BCL11A*, *TCL1A*, *ZFP36L1, NOTCH2*, *FOXO1* or *SOX5* genes (Table S3, Figure S4). Breakpoint localisations were consistent with expected 5’*MYC* in t(8;14)/IGH*::MYC*‐r and 3′*MYC* in t(3;22)/IGL*::MYC*‐r (data not shown).^31^

As expected, *BCL2*‐r was exclusively detected in FL and germinal‐centre DLBCL (GCB‐DLBCL) (*p* = 0.0005). *BCL2*‐r and/or *BCL6*‐r was significantly associated with *REL* gain (*p* = 0.03) and was also enriched in somatic variants of *KMT2D*, *CREBBP* and/or *EP300* (*p* = 0.0062). Conversely, the co‐occurrence of at least two of four cytogenetic markers [+3q/+18q/del(6q)/del(*CDKN2A*)] was more frequently detected in eight of nine non‐GCB DLBCL compared with two of nine GCB‐DLBCL (*p* = 0.004) and was significantly associated with *MYD88* ^*L265P*^, *CD79B* and/or *PIM1* mutation (*p* = 0.0001). Further details are provided in Table S4.

**FIGURE 3:**
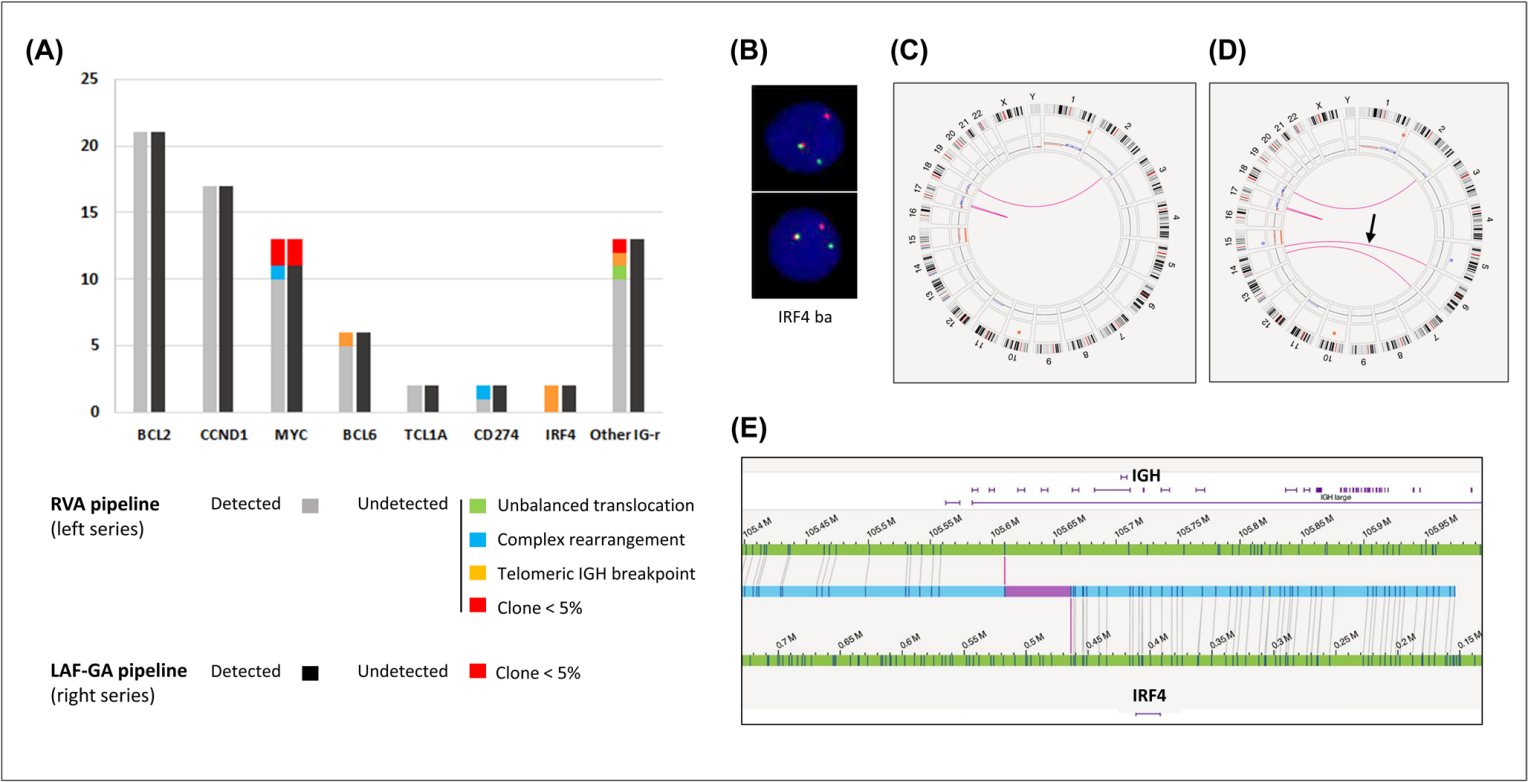
Comparison of low allele fraction‐guided assembly (LAF‐GA) pipeline and rare variant analysis (RVA) pipeline for the detection of immunoglobulin (IG) rearrangements by optical genome mapping (OGM). (A) Number of IG rearrangements detected or not detected by the RVA and/or LAF‐GA pipelines. IG rearrangements are ordered according to their partner gene (X‐axis). The colour code indicates the reason for non‐detection. (B–E) Illustration of the performance of LAF‐GA pipeline in identifying IGH*::IRF4* rearrangements. Case #14, large B‐cell lymphoma with *IRF4* rearrangement. (B) Interphase FISH showing a split with the IRF4 breakapart (ba) probe. (C) Circos plot view using the RVA pipeline, failing to detect the *IRF4* rearrangement. (D) Circos plot view using LAF‐GA pipeline revealing the t(6;14)/IGH*::IRF4* (black arrow). (E) Genome map view of the IGH*::IRF4* rearrangement. The upper and lower green banners represent the 14q32.33/IGH and the 6p25.3/*IRF4* loci, respectively. The central blue banner represents the sample map corresponding to the t(6;14)/IGH*::IRF4* rearrangement. The pink segment indicates the breakpoint region. The circos plot displays four concentric rings: The outermost represents chromosome ideograms; the second ring shows CNVs (gains in blue, losses in red); the third ring shows SVs (deletions in orange, duplications in blue, insertions in green). The innermost pink lines indicate intra‐ and inter‐chromosomal translocations. [Colour figure can be viewed at wileyonlinelibrary.com]

Previous reports have pointed out limitations of OGM in detecting IG translocations using the RVA pipeline.^24^, ^27^ The LAF‐GA pipeline confidently calls 75/77 IG‐r (97%), missing only the two low‐level *MYC*‐r. In contrast, the RVA pipeline detected 64/77 (85%), missing both t(6;14)/IGH*::IRF4*‐r and four dual IG‐r (Figure 3). At a 5% allele frequency, LAF‐GA clearly outperformed RVA for detection of IG‐r, averaging 100%.

### OGM provides diagnostic and prognostic information

**FIGURE 4:**
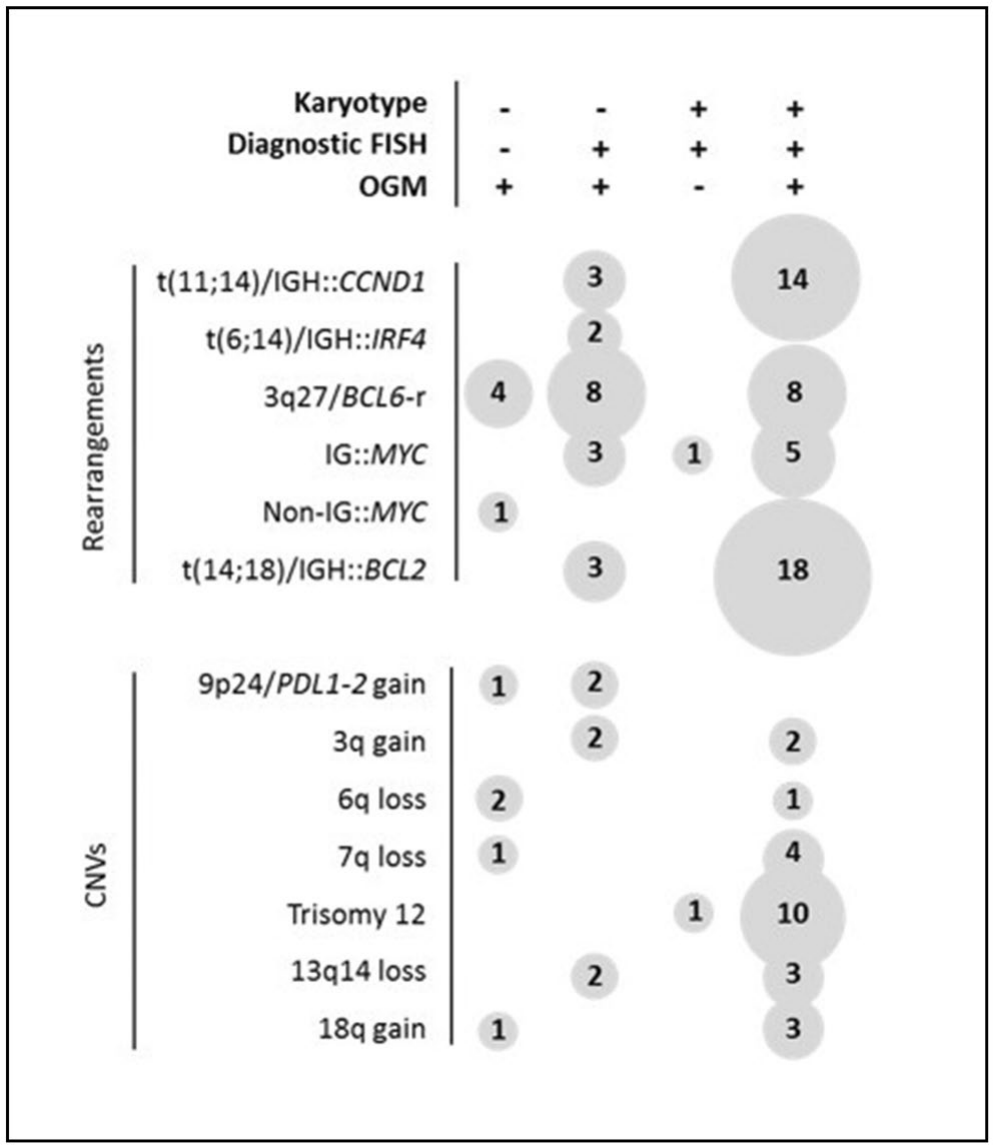
Detection of relevant diagnostic cytogenetic alterations by different techniques (karyotype and/or FISH and/or optical genome mapping (OGM)). Each circle represents a set of rearrangements and copy number variations (CNVs) detected by a unique combination of techniques (columns). The size of each circle is proportional to the number of events (also indicated within each circle). Rows indicate diagnostically relevant rearrangements (top), including cases with multiple rearrangements per patient and CNVs (bottom). The combination of techniques is indicated at the top of the figure: Plus (+) and minus (−) signs denote whether the technique detected or did not detect the corresponding rearrangement or CNV respectively. Combinations that did not match with a diagnostically relevant rearrangement or CNV are not depicted.

OGM identified diagnostically relevant CAs in 99/105 patients (94.2%) compared with 77.1% (81/105 patients) for karyotype alone, 83.6% (82/98 patients) for FISH alone and 89.5% (94/105 patients) for combined CC (Figure 4).

Six cryptic rearrangements (5.7%) targeting *MYC*, *BCL2* and *BCL6* loci were detected exclusively by OGM, including two small insertions (<200 kb) of *MYC* and *BCL2*, whereas breakapart FISH probes returned false‐negative or equivocal patterns (Table 2; Figure S5). Through OGM only, the final diagnosis was refined for three cases.

**TABLE 2:** OGM detects cryptic and complex rearrangements involving diagnostically relevant genes (MYC, BCL2, BCL6).

No. patient	Target gene	FISH results with a breakapart probe	Rearrangement detected by OGM	Involved mechanism (size)	Initial diagnostic orientation	Final integrated diagnosis
#88^a^, ^b^	*MYC*	Negative	ins(14;8)(q32;q24.21q24.21) (IGH*::MYC*)	Insertion (180 kb)	HGBL	BL^c^, ^d^
#1^e^	*MYC*	Equivocal unbalanced	del (8)(q24.21q24.21)inv(8)(q24.21q24.22) t(8;14)(q24;q21.1) non‐IG*::MYC*	Complex pattern (3 Mb)	DLBCL, non‐GCB	HGBL, *MYC/BCL6* ^c^ DLBCL, *MYC/BCL6* ^d^
#56^b^	*BCL2*	Negative	ins(14;18)(q32;q21q21) (IGH*::BCL2*)	Insertion (200 kb)	FL grade 1/2	FL grade 1/2 with *BCL2‐*r^c^ Classical FL with *BCL2‐*r^d^
#76	*BCL6*	Equivocal unbalanced	del (3)(q27.3q27.3) (*LPP::BCL6*)	Deletion (1,1 Mb)	DLBCL, GCB	DLBCL, GCB with *BCL6*‐r^c^, ^d^
#61^e^	*BCL6*	Negative	del (3)(q27.3q27.3) (*LPP::BCL6*)	Deletion (325 kb)	HGBL, NOS	HGBL, *MYC/BCL6* ^c^, ^d^
#85^e^	*BCL6*	Negative	inv(3)(q27.3q27.3)(*EIF4A2::BCL6*)	Inversion (306 kb)	Small B‐cell lymphoma	FL grade 1/2 with *BCL6‐*r^c^ Classical FL with *BCL6*‐r^d^

Abbreviations: BL, Burkitt lymphoma; DLBCL: Diffuse large B‐cell lymphoma; FL, Follicular lymphoma; GCB, Germinal centre B‐cell subtype; HGBL‐MYC/BCL6: High‐grade B‐cell lymphoma with both *BCL6* and *MYC* rearrangements; r, rearrangement.

^a^ Rearrangement detected exclusively by the LAF‐GA pipeline.

^b^ Cases diagnosed by FISH using dual‐fusion FISH probes (see Figure S5).

^c^ The final diagnosis according to the ICC, followed by additional rearrangements identified by OGM.

^d^ The final diagnosis according to the WHO‐HAEM5 classification, followed by additional rearrangements identified by OGM.

^e^ The final diagnosis was established based on OGM findings.

Among 15 patients with non‐informative karyotypes (NIK), OGM successfully revealed the disease‐associated CAs in 11/11 cases (Figure 2), compared with 7/11 cases by diagnostic FISH (Table S3, Figure S6).

Integration of OGM findings into our multidisciplinary approach achieved diagnostic consensus in 102/105 cases. OGM results also resolved the three remaining difficult cases, with conclusions supported by multidisciplinary review: (1) an extra‐nodal DLBCL case (#74) carrying focal losses of *PRDM1*, *CDKN2A* and a 19p13/*SPIB* duplication alongside a *MYD88* ^*L265P*^/*CD79B* co‐mutation; (2) a transformed MZL (#78) case with *BCL6*‐r, trisomy 3, 7 and 12; (3) a peripheral T‐cell lymphoma, not otherwise specified (#100) case with a complex genome including a *TP53* deletion (Figure S7).

Compared with CC, OGM identified additional prognostic markers in four patients. These included a subclonal IGH*::MYC*‐r in a patient with FL (#75) as well as three patients with NIK (two MCL (#57 and #60) and one lymphoplasmacytic lymphoma (#84)) who met the criteria for CK when large CNVs (>10 Mb) were considered (Table S3).

Overall, OGM provided added diagnostic or prognostic value for 10 (9.5%) and four patients respectively.

### OGM can assess genomic complexity

**FIGURE 5:**
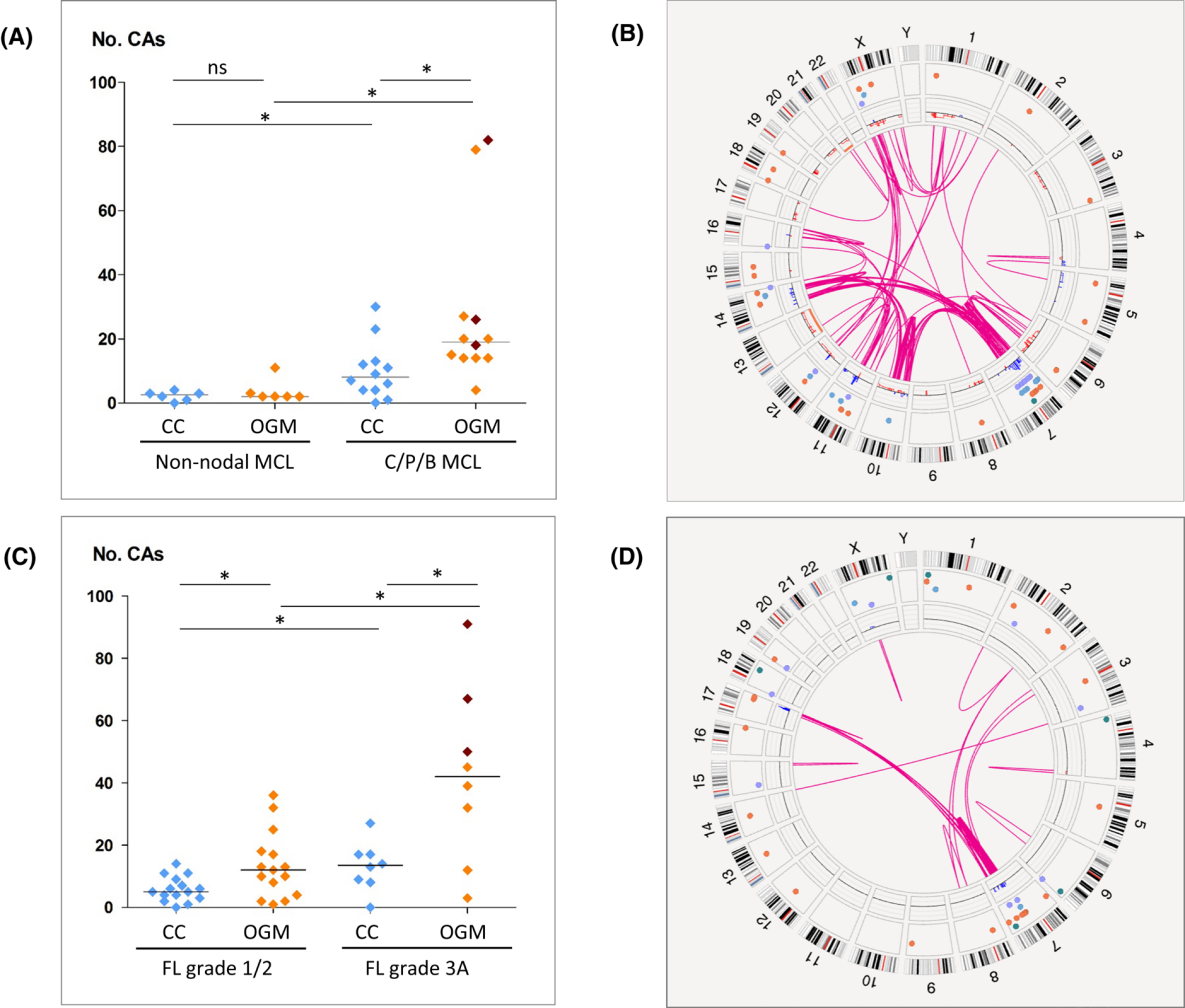
Genomic complexity assessed by optical genome mapping (OGM) in mantle cell lymphoma and follicular lymphoma. (A, C) Number of cytogenetic abnormalities (CAs) detected by classical cytogenetics (CC, blue squares) and OGM (orange and maroon squares) in mantle cell lymphoma (MCL) and follicular lymphoma (FL). Scatter plots show the respective numbers of CAs in classical, pleomorphic and blastoid MCL (C/P/B MCL) compared with non‐nodal MCL (A) and in FL grade 1/2 compared with FL grade 3A (B). Maroon squares indicate the six cases exhibiting a chromoanagenesis pattern. Horizontal grey lines represent medians of each subgroup. ns, not significant. *p < 0.05. (B, D) Circos plot views illustrating complex cytogenomic patterns in MCL (B) and FL (D). (B) Case #10, classical MCL. Highly complex pattern with numerous SVs, CNVs and intra‐ and inter‐chromosomal translocations involving chromosomes X, 1, 6, 7, 10, 11, 12, 14 and 16, consistent with chromoanagenesis. (D) Case #47, FL grade 3A. Complex pattern involving both q arms of chromosomes 7 and 17, including clusterisation of multiple structural variants (SVs)and copy number variations (CNVs), going from one to the next with gains and losses, corresponding to chromoanagenesis. In each circos plot, the outermost ring shows chromosome ideograms, followed by a ring displaying SVs <5 Mb (blue circles for duplications, orange circles for deletions and green circles for insertions) and an inner ring for CNVs (>500 kb) and aneusomies (gains in blue; losses in orange and red). The innermost pink lines represent inter‐ or intra‐chromosomal translocations. [Colour figure can be viewed at wileyonlinelibrary.com]

Using a threshold of ≥10 CAs (size >250 Kbp), as recently used in CLL, 10/11 MCL with CK were also classified as complex by OGM. As previously reported by CMA, genomic complexity was higher in cMCL versus nnMCL and in FL grade 3 versus grade 1/2.^24^, ^32^, ^33^ Consistent with CC, OGM revealed a significantly higher genomic complexity in blastoid, pleomorphic and cMCL compared with nnMCL and in FL grade 3A compared with FL grade 1/2 (Figure 5). All classical, pleomorphic or blastoid MCL cases tested by NGS (10/10) harboured *TP53* and/or *ATM* alterations. Across the whole cohort, 24/25 (96%) *TP53*‐altered cases exhibited a complex genome, whereas 24/41 (58.5%) *TP53* wild‐type cases had a non‐complex genome (*p* < 0.0001).

OGM identified 10 chromoanagenesis patterns, all complex by karyotype and OGM; 6/8 cases assessed by NGS were *TP53*‐altered.

## DISCUSSION

OGM has recently emerged as an effective ‘next‐generation karyotyping’ approach in several HM.^34^, ^35^, ^36^, ^37^, ^38^ Using diverse NHL sample types, including fresh and frozen tissues, we conducted the first large prospective study demonstrating that OGM sensitively detects all diagnostically relevant CAs and can complement or potentially replace CC to improve cytogenomic characterisation of NHL.

We showed that OGM is readily feasible for NHL specimens, integrating fresh and frozen tissue samples into the diagnostic workflow through a streamlined protocol that shortens preparation time for frozen material without compromising data quality or TAT. However, to support the clinical implementation of OGM in NHL, parallel cell culture should be considered to enable a rescue karyotype in cases of OGM failure. Compared with CC, OGM demonstrated a TAT compatible with routine diagnostics.

A retrospective comparison of OGM to CC was recently published by Verhasselt et al., demonstrating a high concordance rate (>90%), added value in NIK and the ability of OGM to uncover additional abnormalities.^28^ Consistent with that study and previous reports in HM, our data confirm that OGM may miss low‐level CNVs (<15%–20%) and SVs (<5%).^22^, ^24^, ^25^, ^28^ Consequently, clinically relevant low‐fraction clones, such as *TP53* deletions, may still be more reliably detected by FISH.^24^, ^39^ Our study further demonstrates that LAF‐GA, applied here in the whole cohort, is particularly effective and therefore recommended in B‐NHL for detecting dual or complex IG‐r and accurately identifying their partner genes. *CD274/PDCD1LG2* (encoding PDL1/PDL2) rearrangements or amplifications have been associated with overexpression in primary mediastinal B‐cell lymphoma and systemic DLBCL.^40^ As checkpoint inhibitor therapy appears to confer clinical benefit in this setting, OGM could represent a reliable method for identifying such patients.^41^, ^42^

Our results highlight the substantial diagnostic impact of OGM, providing crucial information for 9.5% of patients with cryptic events, NIK or otherwise challenging contexts. OGM notably outperformed FISH in detecting cryptic *MYC*, *BCL2* and *BCL6* rearrangements which occur in up to 5% of DLBCL, HGBL and FL.^6^, ^14^, ^16^ In line with our observations, whole genome sequencing studies have shown that half of FISH false‐negative *BCL6*‐r cases correspond to cryptic events involving nearby 3q27 partner genes (e.g. *ST6GAL1*, *EIF4A2* and *LPP*).^6^, ^43^ We also identified a novel HGBL‐MYC/BCL6 case with 11q aberration in an elderly patient. Although HGBL‐11q occurs typically in children or young adults, 11q aberrations have also been described in elderly patients with *MYC‐*, *BCL6*‐ or *BCL2*‐rearranged B‐NHL.^44^ As a stand‐alone assay, OGM not only captured the enhancer‐driven rearrangements but also identified the highest proportion of patients with class‐defining CAs.

Beyond diagnosis, OGM may provide prognostic information. In FL, we previously reported that dual IG::*MYC*‐r/*BCL2*‐r is associated with increased transformation risk and shorter progression‐free survival.^45^ In the present series, OGM also revealed secondary CNVs of known prognostic significance, including *TP53*/*CDKN2A* co‐deletion, associated with adverse outcomes in MCL, DLBCL and FL.^43^, ^46^, ^47^, ^48^, ^49^ In addition, Qu et al. reported that individual submicroscopic CNVs such as 2p16 gain (*REL*, *BCL11A*), 17p13/*TP53* loss and 15q21/*B2M* loss are associated with shorter overall survival in FL.^49^ *MIR17HG* gain, detected here in some samples, has been linked to inferior progression‐free survival in DLBCL.^16^, ^43^ In HGBL‐MYC/BCL2, adverse prognosis may be specifically linked to IG::*MYC*‐r, as opposed to other *MYC* partner genes, although this distinction is rarely assessed in practice as routine FISH assays do not systematically include dual‐fusion probes.^50^, ^51^, ^52^ In contrast, the HGBL‐MYC/BCL6 category remains heterogeneous, including cases with t(3;8)(q27;q24)/*BCL6::MYC*‐r, of unclear outcomes.^53^

OGM also enables the characterisation of complex genomes, which may carry prognostic relevance across B‐NHL.^10^ This is particularly relevant for MCL patients, where karyotyping may fail despite optimised culture conditions.^12^ Genomic complexity in MCL is clinically critical, as CK or complex genome is associated with high‐risk disease and significant therapeutic implications.^7^, ^8^, ^32^, ^54^, ^55^ As previously reported, we confirm that *TP53*+/−*ATM* alteration is significantly enriched in classical, pleomorphic or blastoid MCL variants.^32^ Using a threshold of 10 CAs, as previously applied in CLL, OGM successfully classified MCL cases with complex genomes.^24^ In FL, a high aberration burden, including large CNVs identified by CMA, has been associated with earlier progression and shorter overall survival in FL.^49^, ^56^ Regarding chromoanagenesis, our small sample size limited assessment of its association with *TP53* alterations, although this link has been reported in other HMs.^57^, ^58^

OGM captures the cytogenetic heterogeneity of DLBCL. Recent genomic profiling studies have delineated five to seven molecular subtypes with distinct prognostic implications, defined by specific combinations of mutations, rearrangements and CNVs.^43^, ^59^ The MCD/C5 subtype, characterised by *MYD88/CD79B* co‐mutation, is enriched in *PRDM1*/6q21 and *CDKN2A*/9p21 losses alongside *BCL2*/18q21 and *SPIB*/19q13 gain. In contrast, the EZB/C3 subtype, defined by *BCL2*‐r and *EZH2* mutations, is enriched in alterations of epigenetic modifiers (*KMT2D*, *CREBBP*, *EP300*), *REL* gain and *MI17HG* amplification, resembling FL. MCD/C5 is strongly associated with non‐GCB‐DLBCLs, whereas EZB/C3 is prototypical of GCB‐DLBCL. Despite a limited number of cases, our data suggest that OGM is able to identify these cytogenetic signatures in concordance with molecular alterations. The combined use of targeted NGS and OGM may, therefore, provide an efficient strategy for DLBCL molecular subclassification.

The main limitations of this study are its single‐centre design and moderate cohort size. Rare entities such as HGBL‐MYC/BCL2 or HGBL‐11q were therefore not represented. However, our previous OGM analysis of eight DLBCL cell lines, including five DLBCL/HGBL‐MYC/BCL2 and one HGBL‐11q, detected all expected CAs using the LAF‐GA pipeline.^60^ The absence of CMA was mitigated by extended FISH validation of recurrent CAs. As expected from a high‐resolution technique, OGM identified novel aberrations (e.g. IGH‐r involving *NOTCH2*, *SOX5* or *FOXO1*) requiring further investigation.

In conclusion, this prospective study demonstrates that OGM, performed as a single standardised assay, improves the detection and characterisation of cytogenetic alterations, particularly in cases of NIK or complex diagnostic settings, thereby reducing the need for FISH testing and optimising diagnostic TAT. OGM complements targeted NGS by providing genome‐wide structural insight and surpasses karyotyping and FISH in refining diagnoses, identifying class‐defining rearrangements and prognostic markers. Altogether, our findings illustrate how OGM is well positioned to transform front‐line cytogenetic testing in NHL.

## AUTHOR CONTRIBUTIONS

**Coura Fall:** Formal analysis; validation; writing – original draft; visualization. **Agnès Daudignon:** Formal analysis; writing – review and editing; validation. **Séverine Valmary‐Degano:** Formal analysis. **Clémentine Legrand:** Formal analysis; visualization. **Edouard Bonneville:** Formal analysis; visualization. **Lucile Bussot:** Formal analysis. **Julie Mondet:** Formal analysis. **Sylvie Tondeur:** Formal analysis; visualization. **Pierre Aubert:** Formal analysis; visualization. **Simon Chevalier:** Formal analysis; visualization. **Lysiane Molina:** Formal analysis. **Anouk Emadali:** Formal analysis; visualization. **Hélène Guermouche:** Formal analysis; visualization. **Sylvain Carras:** Conceptualization; writing – review and editing; methodology; investigation; funding acquisition. **Christine Lefebvre:** Conceptualization; investigation; funding acquisition; writing – original draft; supervision; validation; writing – review and editing; methodology. **Jean‐Baptiste Gaillard:** Formal analysis; visualization.

## FUNDING INFORMATION

This work was supported by a grant from the Department of Health Research and Innovation of the Grenoble University Hospital (N° 38RC22.0283, CARTOGHEM), as well as by the ARAMIS Association (*Association pour la Recherche sur les Affections Malignes en Immunologie Sanguine, Grenoble, France*).

## CONFLICT OF INTEREST STATEMENT

The authors have no conflicts of interest to disclose.

## Supporting information


**Figure S1.** Number of CAs by classical cytogenetics and OGM.
**Figure S2.** Feasibility of OGM in routine practice for NHL across all sample types.
**Figure S3.** Illustration of false‐negative CAs by OGM.
**Figure S4.** Identification of rare or novel IG rearrangements of undetermined significance.
**Figure S5.** OGM detected diagnostically cryptic rearrangements.
**Figure S6.** Diagnostic relevance of OGM in cases of non‐informative karyotype.
**Figure S7.** Illustration of OGM and FISH results for the three patients with initial uncertain diagnosis.
**Table S1.** List of FISH probes.
**Table S2.** BED file lymphoma: list of 366 genes and loci.
Table S3.
**Table S4.** Distribution of the main CAs in FL, GCB‐DLBCL, GCB NHL and non‐GCB‐DLBC.

## Data Availability

For original data, please contact clefebvre@chu-grenoble.fr.
