# Supplementary material for: Optical genome mapping improves detection and characterisation of cytogenetic abnormalities in non‐Hodgkin lymphomas
Source: Br J Haematol. 2026 May 24;209(1):31–41. doi: 10.1111/bjh.70575 (PMC13340520; doi:10.1111/bjh.70575)
Supplement: Supplementary file 1 — Figure S1. Number of CAs by classical cytogenetics and OGM. Figure S2. Feasibility of OGM in routine practice for NHL across all sample types. Figure S3. Illustration of false‐negative CAs by OGM. Figure S4. Identification of rare or novel IG rearrangements of undetermined significance. Figure S5. OGM detected diagnostically cryptic rearrangements. Figure S6. Diagnostic relevance of OGM in cases of non‐informative karyotype. Figure S7. Illustration of OGM and FISH results for the three patients with initial uncertain diagnosis. Table S1. List of FISH probes. Table S2. BED file lymphoma: list of 366 genes and loci. Table S3. Table S4. Distribution of the main CAs in FL, GCB‐DLBCL, GCB NHL and non‐GCB‐DLBC. [file BJH-209-31-s001.zip › bjh70575-sup-0001-Supinfo/bjh70575-sup-0001-Supinfo1@Supplemental material revised Lefebvre.docx]

**Supplemental material**

**Optical genome mapping improves detection and characterisation of cytogenetic abnormalities in non-Hodgkin lymphomas.**

Coura Fall, Agnès Daudignon, Séverine Valmary-Degano, Julie Mondet, Lucile Bussot, Lysiane Molina, Simon Chevalier, Edouard Bonneville, Pierre Aubert, Sylvie Tondeur, Clémentine Legrand, Jean-Baptiste Gaillard, Hélène Guermouche, Anouk Emadali, Sylvain Carras, Christine Lefebvre.

**Supplemental methods**

**Cytogenetic analyses**

Conventional karyotyping was performed using unstimulated short-term cultures (17 h; 30 to 50.10^6^ cells) without mitogens, and long-term cultures (72 h; 20.10^6^ cells) stimulated with CpG-oligonucleotide, DSP30, and interleukin 2. Metaphases were analysed after R-banding. A karyotype was considered normal when no abnormalities were detected on 20 metaphases. A non-informative karyotype included normal karyotypes, unsuccessful cultures, and karyotypes with isolated X or Y loss. Complex karyotype (CK) was defined by the presence of more than three clonal cytogenetic abnormalities (CAs), consistent with the current definition for mantle cell lymphoma.

For FISH analyses, 100 to 200 nuclei were evaluated per slide and, when possible, 10 metaphases were analysed. Images were captured and analysed using a Metafer system (Metasystems). Five samples underwent diagnostic FISH on FFPE tissue sections in parallel to FISH on cytogenetic pellets. For FISH on FFPE, the detection threshold applied was 10%.

**Optical genome mapping**

**Cell preparation**

All pellets for OGM were conditioned and cryopreserved at -80 °C within 24 h of sample collection.

Fresh lymphoma tissue from our dedicated lymphoma circuit was aseptically dissociated for cell culture and viability assessment. When viability exceeded 50%, two dry pellets containing 2.10⁶ cells each were cryopreserved for OGM. This 50% threshold was defined during preliminary testing as the minimal requirement to obtain ultra-high-molecular-weight (UHMW) DNA of suitable quality.

For peripheral blood, bone marrow and body fluids, leftover material from cell culture was used to cryopreserve two dry pellets (1.5 10⁶ cells each) at -80°C.

For frozen tissue, we developed and validated a simplified, rapid procedure to obtain a cell suspension compatible with UHMW DNA extraction. Approximately 5-8 mg of frozen tissue were thawed on a sterile chilled surface, manually fragmented, and gently crushed in a cold microtube using a pellet pestle. The material was gently washed in 5 mL of cold dissociation buffer (1% EDTA in DPBS, Panbiotech, Germany), and then filtered through a 70-µm sieve. After centrifugation and removal of the supernatant, cells were resuspended in Bionano cold stabilization buffer with RNase-A. Cells were then processed alongside other samples for UHMW DNA extraction.

**DNA extraction and direct enzymatic labelling**

UHMW genomic DNA was manually extracted from cryopreserved cell pellets (PB, BM, body fluid or tissue) using the Prep SP-G2 Frozen Cell Pellet DNA Isolation kit, following the manufacturer’s instructions (Bionano Genomics, San Diego, CA, USA). Briefly, samples were lysed and digested with Proteinase K, RNase A, and Lysis Binding buffer. DNA was precipitated with isopropanol and bound to a Nanobind magnetic disk, eluted and quantified using Qubit Broad Range dsDNA kits (ThermoFisher Scientific). DNA was homogenised for 24-48 h at room temperature.

Labelling was performed in line with the manufacturer’s protocol (Bionano Prep DLS-G2, Bionano Genomics, San Diego, CA, USA). A standard Direct Label Enzyme 1 (DLE-1) reaction was carried out using 750 ng of UHMW DNA targeting the CTTAAG motif with DL-green fluorophores. After enzymatic digestion (PureGene Proteinase K, Qiagen), and adsorption for fluorophore cleanup, labelled DNA was counterstained (blue backbone staining), quantified with Qubit High Sensitivity dsDNA kits (ThermoFisher Scientific, USA), and prepared for loading.

**Data collection and quality metrics**

Labelled DNA molecules were loaded onto a Saphyr G3.3 chip and imaged on the Saphyr instrument (Bionano Genomics). Individual linearized DNA molecules were displaced through nanochannels by electrophoresis. Multiple cycles were run until 1600 Gb of data had been collected per sample, reaching ~300x genome coverage.

Quality metrics were evaluated for all samples. To be valid, OGM analysis requires an appropriately labelled DNA length (N50) exceeding 230 kbp (for molecules >150 kbp with ≥ 9 labelled sites), a label density (LD) of 14 to 17 labels per 100 kbp, and a map rate (proportion of molecules aligned to the reference genome) greater than 70%. These thresholds support the detection of CAs at low level fraction (~5%). Overall, 95% of samples met the recommended quality criteria (Supplemental Figure 1). The median value of LD was 15.7 [12.9 – 18.5]. We also assessed two relevant labelling quality parameters: The Positive Label Variance (PLV) which corresponds to the percentage of labels absent in reference (expected values < 10%) and the Negative Label Variance (NLV) which indicates the percentage of reference labels absent in molecules (expected value < 15%). The median values and ranges [min – max] of PLV were 4.8% [1.6 – 11]. The median values and ranges [min – max] of NLV were 8.4% [5.2 – 19]. Five elevated NLV values (17 - 19%) were associated with lower LD (~13) but with a final effective coverage > 300x, resulting in successful analyses. Five fresh tissue and three BM samples showed one parameter below its threshold value, but were nevertheless successfully analysed.

**OGM variant calling**

Captured images were converted into barcode patterns and aligned to the corresponding human Genome Reference Consortium (GRCh38).

Genome analyses were performed using both the Rare Variant Analysis (RVA) pipeline and the Low Allele Fraction Guided Assembly (LAF-GA) pipeline, each detecting structural variants (SVs) and copy number variants (CNVs). Both RVA and LAF-GA pipelines were systematically applied to all samples.

The RVA pipeline is commonly used to identify SVs occurring at a low allele fraction (about 5%) in tumour samples. The principle of the RVA pipeline is that it detects SVs based on differences between labelling patterns between the sample and the GRCh38 assembly. The first step is to compare the molecules to the reference and to retain those that are misaligned. Then, a consensus map file (*.cmap) is generated with the cluster of molecules exhibiting the same misalignment (detection of SV in this case). Finally, each *.cmap is realigned to the GRCh38 to produce the final SV call.

The LAF-GA pipeline combines low-allele fraction detection with whole-genome assembly to enhance identification of fine or subtelomeric SVs at low allele fraction (~5%).

LAF-GA is a variant of the de novo assembly pipeline. The de novo assembly pipeline is commonly used to identify constitutional alterations. It consists of constructing a de novo assembly and then comparing the resulting assembly with a human reference genome. The Guided Assembly corresponds to a hybrid approach of de novo and RVA pipelines: LAF-GA pipeline uses a reference genome instead of draft consensus maps as the seed for extension and refinement. LAF-GA includes an initial step of reconstructing a de novo assembly, producing consensus maps that are subsequently refined through extension and merging stages. Then SVs are detected by aligning refined maps to the reference genome. The principal advantage of Guided Assembly (GA) is that it performs a full assembly of the entire genome, rather than a targeted assembly around putative SVs as performed by RVA. This enables GA to provide more accurate VAF estimation for low-frequency variants and to better identify complex or subtelomeric SVs.

A coverage-based algorithm (embedded in both pipelines) was also used to detect CNVs >500 kbp and aneuploidies by quantifying deviations in label coverage relative to baseline copy number.

**Data analysis and filtering**

RVA and LFA-GA data were analysed using Bionano Access software (Tools version 1.8.1, Bionano Genomics, San Diego, CA, USA). For downstream analysis, CNVs ≥10 Mb were classified as large (detectable by conventional cytogenetics) and CNV ≤10 Mb were classified as submicroscopic.

In order to select aberrations with potential diagnostic relevance, we further filtered all confident CNVs and SVs using a custom-made Browser Extensible Data (BED) file. This file was derived from a collaborative review of the literature, performed by seven cytogeneticists with expertise in NHL from the FrOGG group (French speaking OGM Group from the GFCH, Groupe Francophone de Cytogénétique Hématologique). This BED file was designed to include not only diagnostically relevant genes and rearrangements of B-cell lymphomas (as described in the section “Mature B-cell disorders” of the WHO classification of hematolymphoid tumours and the International Consensus Classification) but also recurrent gains and losses reported in B-cell lymphomas.

This file contains 366 genes and loci, including “large genes” to capture breakpoints located far from the 5’ or 3’ ends of critical genes, as described in the literature. These “large genes” include: *BCL2, BCL6, MYC, CCND1, CCND2, CCND3, CD274, CDK6, IRF4, FOXP1, MALT1, PAX5, BCL3, TCL1A* (Supplemental Table 2). The final list was validated by the FrOGG working group for B-NHL.

The filtering workflow is shown in Supplemental Figure 1. The first steps applied a size threshold (≥500 kb for CNVs, no size threshold for SVs) and recommended confidence scores for aberration calling. Labelling polymorphisms were excluded using the Bionano database of 285 healthy individual genomes; variants in highly polymorphic regions were tagged as “masked” and not analysed, but were still available as output.

At the second step: for SVs, an arbitrary minimal size of 250 kbp was used to facilitate FISH confirmation. SVs were additionally filtered at a VAF ≥5%. Among translocations, artefacts were excluded using recommended rules: insufficient labels (<10) on either side of the breakpoint, ambiguous labelling patterns, low supporting molecules and low confidence score (<0.5). The BED file ‘B-cell lymphomas’ was finally applied to detect small (<250 kbp) losses and/or gains and SVs of interest.

Somatic SVs, CNVs and aneuploidies were filtered after RVA and LAF-GA pipeline, using the following quality settings:

SV filters:

Feature SV overlap precision (Kbp) 12

SV masking filter Non-masked variant SVs

VAF filter 0-1

Self-molecule count 5

% in control database 0

% in control databse for enzyme 0

SV chimeric score All SVs

Found in self molecules yes

Overlap genes All

SV type confidence (insertion, deletion,

Inversion, duplication, intra-fusion, Recommended

Inter-translocation)

CNVs filters:

Feature CNV overlap precision (kbp) 500

Copy number type All

Copy number confidence 0.99

Copy number min size (bp) 500 000

Copy number masking filter Non masked CNV

Aneuploidy filters:

Aneuploidy type All

Aneuploidy confidence 0.95

For CNVs and aneuploidies, visual inspection of whole-genome profiles allowed inclusion of large sub-clonal CNVs (>20 Mb). Twenty-one aneuploidies or CNVs that were detectable by OGM only after visual inspection were successfully called after relaxing the filtering criteria (confidence score set to “ALL”). These sub-clonal CNVs were retained as true alterations when confirmed by FISH. For comparison with conventional cytogenetics, adjacent CNV segments were into single CA.

**Concordance Analysis**

To compare CAs identified by CC and OGM at the aberration level, adjacent CNV segments detected by OGM were grouped as a single CA. Translocations identified by karyotyping and described by OGM with closely spaced breakpoints were considered concordant.

Genomic complexity

A pivotal study assessing genomic complexity by OGM in CLL demonstrated that OGM could identify complex genomes using a threshold of ≥10 abnormalities (>100 kb) per case (Puiggros et al., 2022). The 100 kb threshold corresponded to the resolution of chromosomal microarray. In the present study, we selected a higher minimum size of 250 kb, as it approximates the size of the FISH probes used to validate additional CAs detected by OGM. Accordingly, genomic complexity was defined as ≥10 chromosomal abnormalities with a size >250 kb.

Criteria for chromoanagenesis

Chromoanagenesis refers to catastrophic one-step events characterized by numerous genomic abnormalities, including oscillating CNV and inter- and/or intra-chromosomal rearrangements involving one or more chromosomes. In our study, chromoanagenesis was retained after review of all highly complex genomes by three cytogeneticists (C.L., H.G., J-B.G.) based on one of the following patterns: i) Presence of at least 3 clustered CNVs linked to SVs on the same chromosome; ii) Involvement of at least three chromosomes with a minimum of four “chained” interchromosomal SVs connected by shared CNV breakpoints.

**Molecular biology**

Targeted next-generation sequencing was performed in 65 patients, using a 73-gene panel. Additional diagnostic analyses included: B/T clonality (n = 5), Reverse Transcription-Multiple Ligation-dependent Probe Sequencing (n = 25), and IGHV mutational status in CLL (n = 13). RNA sequencing was performed in one case to confirm a *SEC31A::JAK2* fusion transcript identified by OGM in a peripheral T-cell lymphoma (data not shown).

**Supplemental tables**

**Supplemental Table 1: List of FISH probes**

| Target (location) | Probe type | Manufacturer |
| --- | --- | --- |
| ATM/TP53 (11q22/17p13) | Covering probes | Metasystems* |
| B2M (15q21) | Covering probe | Empire genomics* |
| BCL2 (18q21) | Breakapart | Cytocell* |
| BCL6 (3q27) | Breakapart | Cytocell* |
| CCND1 (11q13) | Breakapart | Cytocell* |
| CCND2 (12p13) | Breakapart | Empire genomics* |
| CCND2 (12p13) | Covering probe | Empire genomics* |
| CDK6 (7q21) | Breakapart | Empire genomics* |
| CDKN2A (9p21) | Covering probe | Metasystems* |
| CDKN2C/CKS1B (1p32/1q21) | Covering probes | Metasystems* |
| CIITA (16p13) | Breakapart | Empire genomics* |
| CUX1/EZH2 (7q22/7q36) | Covering probes | Metasystems* |
| DLEU1-2 (13q14) | Covering probe | Cytocell* |
| EGR1/RPS14 (5q31/5q33) | Covering probes | Metasystems* |
| Enhancers IGK (2p11) | Covering probes | Empire genomics* |
| Enhancers IGL (22q11) | Covering probes | Empire genomics* |
| EVI-1(3q26) MECOM | Breakapart | Metasystems* |
| FOXO1 (13q14) | Breakapart | Empire genomics* |
| IGH (14q32) | Breakapart | Cytocell* |
| IGH/BCL2 | Dual fusion | Metasystems* |
| IGH/CCND1 | Dual fusion | Metasystems* |
| IGH/CCND3 | Dual fusion | Cytocell* |
| IGH/FGFR3 | Dual fusion | Metasystems* |
| IGH/MYC | Dual fusion | Metasystems* |
| IGK (2p11) | Breakapart | Cytocell* |
| IGL (22q11) | Breakapart | Cytocell* |
| IRF4 (6p25) | Breakapart | Metasystems* |
| JAK2 (9p24) | Breakapart | Metasystems* |
| MALT1 (18q21) | Breakapart | Cytocell* |
| MIR17HG (13q31) | Covering probe | Empire genomics* |
| MYB (6q23) | Breakapart | Zytovision |
| MYC large (8q24) | Breakapart | Cytocell* |
| NOTCH2 (1p12) | Breakapart | Empire genomics* |
| NUP98 (11p15) | Breakapart | Metasystems* |
| PDGFRB (5q32) | Breakapart | Cytocell* |
| PD-L1 (9p24) | Breakapart | Empire genomics* |
| PD-L1/cen 9 | Covering probe | Empire genomics* |
| PRDM1 (6q21) | Covering probe | Empire genomics* |
| PTPRD (9p24) | Covering probe | Empire genomics* |
| PTPRT/MYBL2 (20q12/20q13) | Covering probes | Cytocell* |
| REL (2p16) | Covering probe | Empire genomics* |
| SEC63/MYB (6q21/6q23) | Covering probe | Metasystems* |
| SOX5 (12p12) | Breakapart | Empire genomics* |
| SPIB (19q13) | Covering probe | Empire genomics* |
| TCF3 (19p13) | Breakapart | Cytocell* |
| TCL1A (14q32) | Breakapart | Metasystems* |
| TET2 (4q24) | Covering probe | Metasystems* |
| TNFAIP3 (6q23) | Covering probe | Empire genomics* |
| TNFRSF14 (1p36) | Covering probe | Empire genomics* |
| TP53/17cen | Covering probe | Cytocell* |
| TP63 (3q28) | Breakapart | Empire genomics* |
| ZFP36L1 (14q24) | Covering probe | Empire genomics* |
| 11q aberration | Gain/Loss | Zytovision* |

**Supplemental Table 2: BED file lymphoma: list of 366 genes and loci**

| *ACTB* | *CDKN2B* | *H2AC7* | *MALT1* | *PIM1* | *SPRY2* |
| --- | --- | --- | --- | --- | --- |
| *APC* | *CDKN2C* | *H2AC11* | *MALT1-large* | *PKM* | *SRSF3* |
| *ARID1A* | *CDKN3* | *H2BC6* | *MAP2K1* | *PKN3* | *ST6GAL1* |
| *ARID1B* | *CHD2* | *H2BC7* | *MAP3K14* | *PLA2G4B* | *STAG3L4* |
| *ARID2* | *CHD8* | *H2BC12* | *MBNL1* | *PLEKHF2* | *STAT3* |
| *ARID3A* | *CIITA* | *H3C4* | *MCL1* | *PLEKHG1* | *STAT6* |
| *ARID4A* | *CNPY3* | *H4C4* | *MCRS1* | *PLEKHG5* | *SUPT3H* |
| *ARID4B* | *CREBBP* | *H4C9* | *MDFIC* | *POLG* | *SVIL* |
| *ASAP1* | *CSF1R* | *HIVEP1* | *MDM2* | *POLR2J* | *SWAP70* |
| *ATM* | *CUL4A* | *HIVEP2* | *MDM4* | *POT1* | *TAP1* |
| *ATRX* | *CUX1* | *HLA-A* | *MEF2B* | *POU2AF1* | *TBL1XR1* |
| *BACH2* | *CXCR4* | *HLA-B* | *MEF2D* | *POU2F2* | *TCF3* |
| *B2M* | *DAZAP1* | *HLA-C* | *METTL1* | *PPP2R2D* | *TCF4* |
| *BCL10* | *DDX3X* | *HMGA1* | *MGA* | *PARK7* | *TCL1A* |
| *BCL11A* | *DEAF1* | *HNRNPA1* | *MIR142* | *PRDM1* | *TCL1A-B-large* |
| *BCL2* | *DFFB* | *HNRNPC* | *MIR15A* | *PRDM16* | *TERT* |
| *BCL2-large* | *DLEU1* | *HNRNPD* | *MIR16-1* | *PRKCB* | *TET2* |
| *BCL2L10* | *DLEU2* | *HNRNPF* | *MIR17HG* | *PTEN* | *TFAP4* |
| *BCL2L11* | *DLEU7* | *HNRNPH1* | *MIR29A* | *PTK2* | *TFPT* |
| *BCL3* | *DMD* | *HSP90AA1* | *MIR29B1* | *PTPN1* | *TFRC* |
| *BCL3-large* | *DMRT1* | *HSP90AB1* | *MKI67* | *PTPRC* | *TGM7* |
| *BCL6-large* | *DOCK2* | *IBTK* | *MLH3* | *PTPRD* | *TJP3* |
| *BCL6* | *DTX1* | *ID3* | *MSL2* | *PVT1* | *TLR2* |
| *BCL7A* | *EBF1* | *IGH* | *MTAP* | *RAD52* | *TMEM25* |
| *NBEAP1* | *EEF1A1* | *IGK* | *MUTYH* | *RAD54L* | *TMEM30A* |
| *BCLAF1* | *EIF4A2* | *IGL* | *MYB* | *RASGEF1A* | *TNFAIP3* |
| *BCOR* | *EP300* | *IGLL5* | *MYC* | *RB1* | *TNFRSF10A* |
| *BIRC3* | *EPHA7* | *IKBKB* | *MYC-large* | *RBM17* | *TNFRSF10B* |
| *BLNK* | *ERCC5* | *IKZF1* | *MYCN* | *RCOR1* | *TNFRSF12A* |
| *BMI1* | *ETS1* | *IL21R* | *MYD88* | *REL* | *TNFRSF14* |
| *BRAF* | *ETV6* | *IL6R* | *NACA* | *RELN* | *TNFRSF25* |
| *BRCA1* | *EWSR1* | *IL7* | *NAV1* | *RFTN1* | *TNFRSF9* |
| *BRCA2* | *EZH2* | *ING1* | *NCOR2* | *RFX7* | *TOX* |
| *BRIP1* | *FAF1* | *IRAG2* | *NECTIN2* | *RHOA* | *TP53* |
| *BTG1* | *FANCC* | *IRF2* | *NFATC1* | *RHOH* | *TP53BP1* |
| *BTG2* | *FANCG* | *IRF2BP2* | *NFkB2* | *RNF128* | *TP63* |
| *CARD11* | *FANCL* | *IRF4* | *NFKB2* | *RPL5* | *TP73* |
| *DNAI7* | *FAS* | *IRF4-large* | *NFKBIA* | *RPS15* | *TRAF3* |
| *CASP3* | *FAT1* | *IRF8* | *NFKBIE* | *RUNX2* | *TRIM13* |
| *CCDC69* | *FBXO11* | *IRS4* | *NFKBIZ* | *S1PR2* | *TRIM33* |
| *CCND1* | *FBXW7* | *ITPKB* | *NLRP8* | *SAMHD1* | *TRRAP* |
| *CCND1-large* | *FCGR2B* | *JAK1* | *NOTCH1* | *SEL1L3* | *UBB* |
| *CCND2* | *FER1L6* | *JAK2* | *NOTCH2* | *SETD1B* | *UBE2O* |
| *CCND2-large* | *FHIT* | *JAK3* | *NOTCH3* | *SETD2* | *UBR5* |
| *CCND3-large* | *FLI1* | *KCNQ1* | *NOTCH4* | *SGK1* | *UGGT2* |
| *CCND3* | *FLNC* | *KDM6A* | *NPHP4* | *SHH* | *USP7* |
| *CCNG1* | *FOXC1* | *KDM4C* | *NPM1* | *SIN3A* | *VOPP1* |
| *CD274* | *FOXO1* | *KLF2* | *NRAS* | *SLC1A5* | *VRK2* |
| *CD44* | *FOXO3* | *KLHL14* | *OSMR* | *SLC9B1* | *WWP1* |
| *CD53* | *FOXP1* | *KLHL6* | *P2RY8* | *SMARCA1* | *XPO1* |
| *CD58* | *FOXP1-large* | *KMT2C* | *PAFAH1B2* | *SMARCA4* | *XPO4* |
| *CD70* | *FOXP2* | *KMT2D* | *PARP1* | *SMC4* | *YY1AP1* |
| *CD74* | *GAPDH* | *KRAS* | *PARP2* | *SCN4A* | *ZCCHC7* |
| *CD79A* | *GAS5* | *LCOR* | *PAX5-large* | *SNHG5* | *ZEB2* |
| *CD79B* | *GNA13* | *LCP1* | *PAX5* | *SOCS1* | *ZNF292* |
| *CDC73* | *GNB1* | *LNP1* | *PCBP1* | *SOCS4* | *ZNF608* |
| *CDK4* | *GPS2* | *LPP* | *PCNX2* | *SOCS6* | *TRAF3* |
| *CDK6-large* | *GRB2* | *LRP1B* | *PCSK7* | *SOX5* | *TRB* |
| *CDK6* | *GRHPR* | *LRP8* | *PDCD1LG2* | *SP140* | *TRD* |
| *CDKN1A* | *GTF2H3* | *LYN* | *PDE4DIP* | *SPEN* | *TRG* |
| *CDKN1B* | *H1-4* | *MAD2L2* | *PDZK1P1* | *SPI1* | *11q23.3 gain* |
| *CDKN2A* | *H2AC6* | *MALAT1* | *PIK3CA* | *SPIB* | *11q24.1 loss* |

**Supplemental Table 3: see excel file**

**Supplemental Table 4: Distribution of the main CAs in FL, GCB-DLBCL, GCB NHL and non-GCB-DLBC.**

|  | **FL**  **(n = 23)** | **GCB-DLBCL**  **(n = 9)** | **GCB NHL (FL + GCB-DLBCL)**  **(n = 32)** | **Non-GCB DLBCL**  **(n = 9)** | **p** |
| --- | --- | --- | --- | --- | --- |
| **Cytogenetic abnormality by OGM:** | | |  | | |
| +X/+Xq | 8 | 2 | 10 | 4 | ns |
| del(1p36)/TNFRSF14 | 3 | 2 | 5 | 1 | ns |
| dup(1q) | 6 | 2 | 8 | 2 | ns |
| REL (2p16) gain | 10 | 4 | 14 | 1 | 0.07 |
| +3/+3q | 2 | 2 | 4 | 6 | **0.002** |
| del(6q) | 9 | 2 | 11 | 4 | ns |
| +7 | 6 | 2 | 8 | 5 | ns |
| MYC (8q24) gain | 6 | 2 | 8 | 3 | ns |
| del(9p21)/CDKN2A | 6 | 6 | 12 | 7 | **0.032** |
| +12 | 7 | 2 | 9 | 1 | ns |
| del(17p13)/TP53 | 3 | 1 | 4 | 2 | ns |
| +18q/+18 | 6 | 1 | 7 | 6 | 0.06 |
| BCL2-r | 18 | 3 | 21 | 0 | **0.0005** |
| BCL6-r | 9 | 4 | 13 | 4 | ns |
| Complex genome | 15 | 6 | 21 | 7 | ns |
| +3q/+18q/del(6q)/del(CDKN2A): minimum two of four * | 4 | 2 | 6 | 8 | **0.004** |
| **Variants by targeted NGS:** | | |  | | |
| N tested | **n = 12** | **n = 7** | **n = 19** | **n = 8** |  |
| KMT2D | 8 | 4 | 12 | 1 | **0.002** |
| CREBBP | 5 | 3 | 8 | 0 | **0.028** |
| EP300 | 3 | 2 | 5 | 1 | ns |
| KMT2D+/-CREBBP+/-EP300 | 8 | 5 | 13 | 2 | **0.038** |
| Histones H1/H2 | 5 | 2 | 7 | 1 | ns |
| BCL2-mutated § | 5 | 0 | 0 | 0 | / |
| MYD88^L265P^ | 0 | 0 | 0 | 4 | / |
| CD79B | 0 | 0 | 0 | 3 | / |
| PIM1 | 1 | 0 | 1 | 3 | / |
| MYD88 ^L265P^+/-CD79B+/-PIM1 * | 1 | 0 | 1 | 8 | **<0.0001** |

The frame in bold indicates the groups being compared and the statistical significance.

* comparison performed between GCB-DLBCL and non-GCB-DLBCL

§ all five BCL2-mutated cases have a BCL2-r

**Supplemental Figures**

**
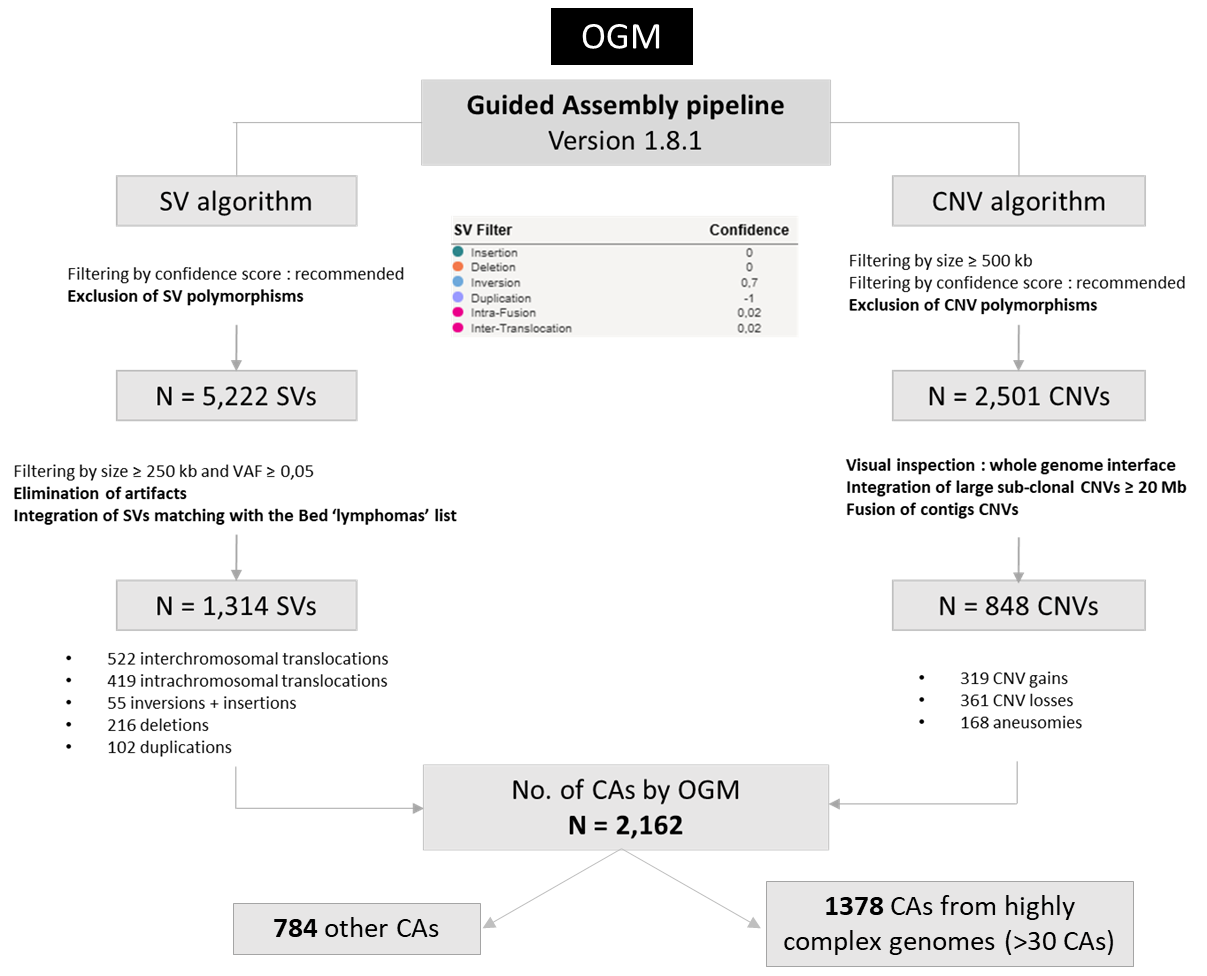
**

**Supplemental Figure 1. Number of CAs by classical cytogenetics and OGM**.

Flow diagram showing the sequence of filters used to detect SVs and CNVs. By OGM, a total of 7,613 CAs were initially called, after filtering, 2,162 CAs were retained. Of those, 1,378 CAs (63.7%) were part of highly complex genomes (≥30 CAs per sample) detected in 23 patients, and thus difficult to compare with CC. A total of 511 CAs detected by OGM were compared to CC. Bold text denotes manual interventions.


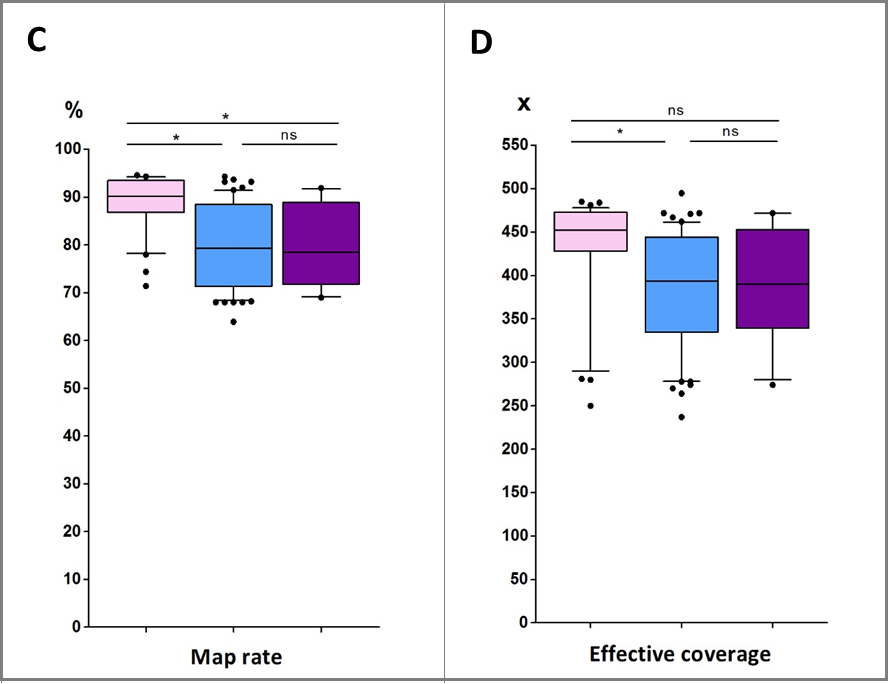

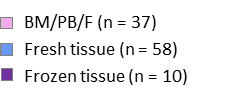

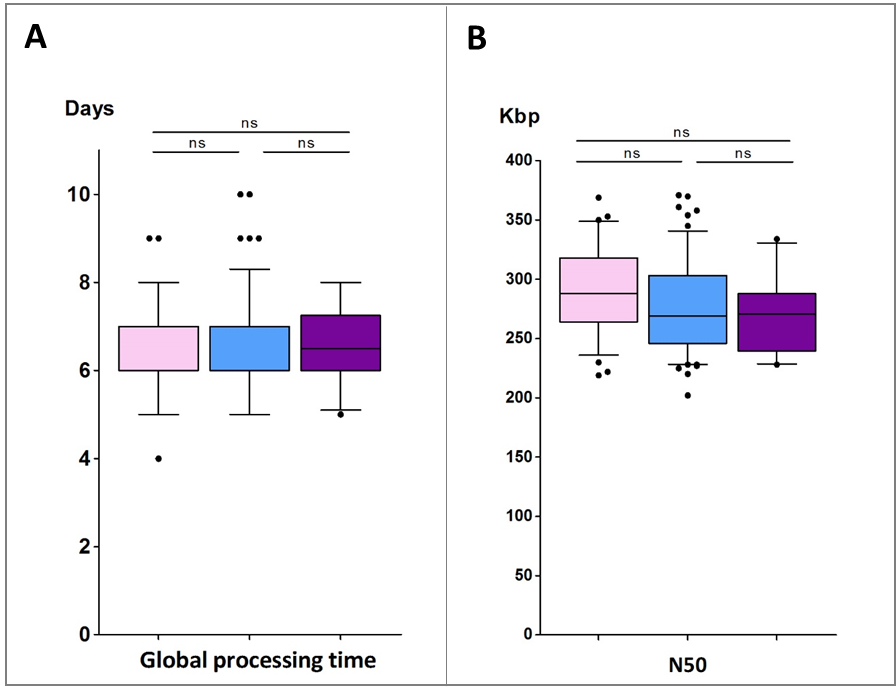


**Supplemental Figure 2. Feasibility of OGM in routine practice for NHL across all sample types.**

(A) Boxplot of total processing time stratified by sample type. Bone marrow aspiration (BM), peripheral blood (PB), and ascites fluid (F) samples (BM/PB/F) are shown in light pink; fresh tissue samples in light blue; and frozen tissue samples in purple. (B, C, D) Boxplots of post-analytic quality metrics: (B) N50 length of extracted and labelled DNA molecules (median: 275 Kbp; recommended minimum: 230 Kbp); (C) Effective genome coverage (median: 425×; recommended minimum: 300×); (D) Map rate (median: 86%; recommended minimum: 70%). ns, not significant. *, p<0.05.

**
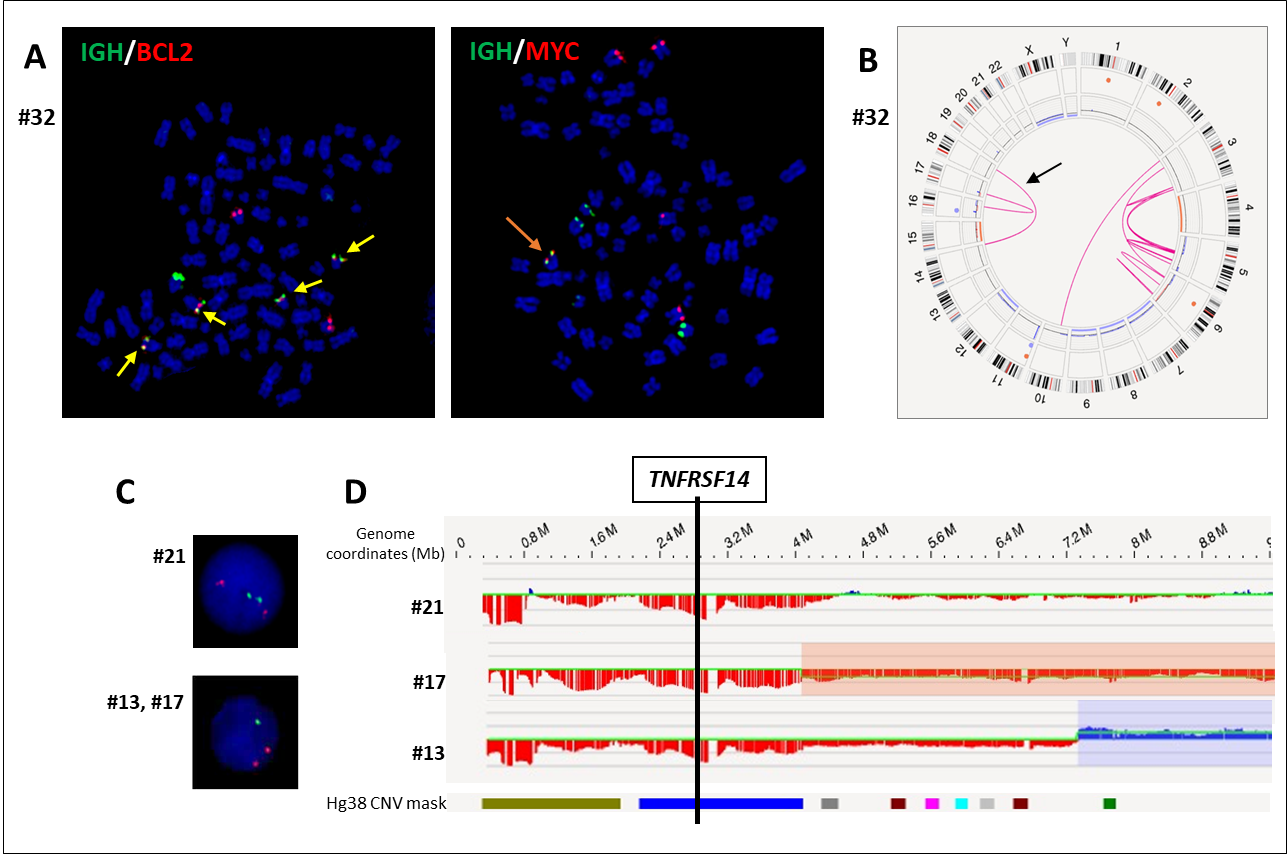
**

**Supplemental Figure 3. Illustration of false-negative CAs by OGM.**

(A) Patient #32, Follicular lymphoma with a tetraploid karyotype. Metaphase FISH using a dual fusion IGH/BCL2 probe revealing two copies of the IGH::*BCL2* rearrangement (yellow arrows); the dual fusion IGH/MYC probe revealed a cryptic *MYC* insertion in a single IGH locus (orange arrow).(B) OGM Circos plot detects IGH::*BCL2* rearrangement (black arrow) but failed to detect IGH::*MYC* rearrangement. (C) Analysis of the *TNFRSF14* locus by FISH compared to OGM. Patient #21 interphase FISH analysis showed a normal profile with two copies of *TNFRSF14* (two green signals for the *TNFRSF14* locus and two red signals for the REL locus, as control), whereas patients #17 and #13 displayed loss of *TNFRSF14* (only one green signal). (D) Genome browser views from OGM for the telomeric 1p36.33 region (0 – 8.8 Mb) for the same patients (#21, #17, and #13), all showing similar OGM patterns from coordinate 0.4 to 4.2 Mb. These similarities are attributed to polymorphisms affecting DNA labelling, which makes the 1p36.33 region difficult to interpret using OGM. For each sample, vertical red bars indicate a deletion pattern whereas blue bars indicate a gain pattern. At the bottom, coloured boxes correspond to polymorphic labelling segments within the 1p36.33 region (Hg38 CNV mask). The vertical black bar marks the location of the *TNFRSF14* gene.

**
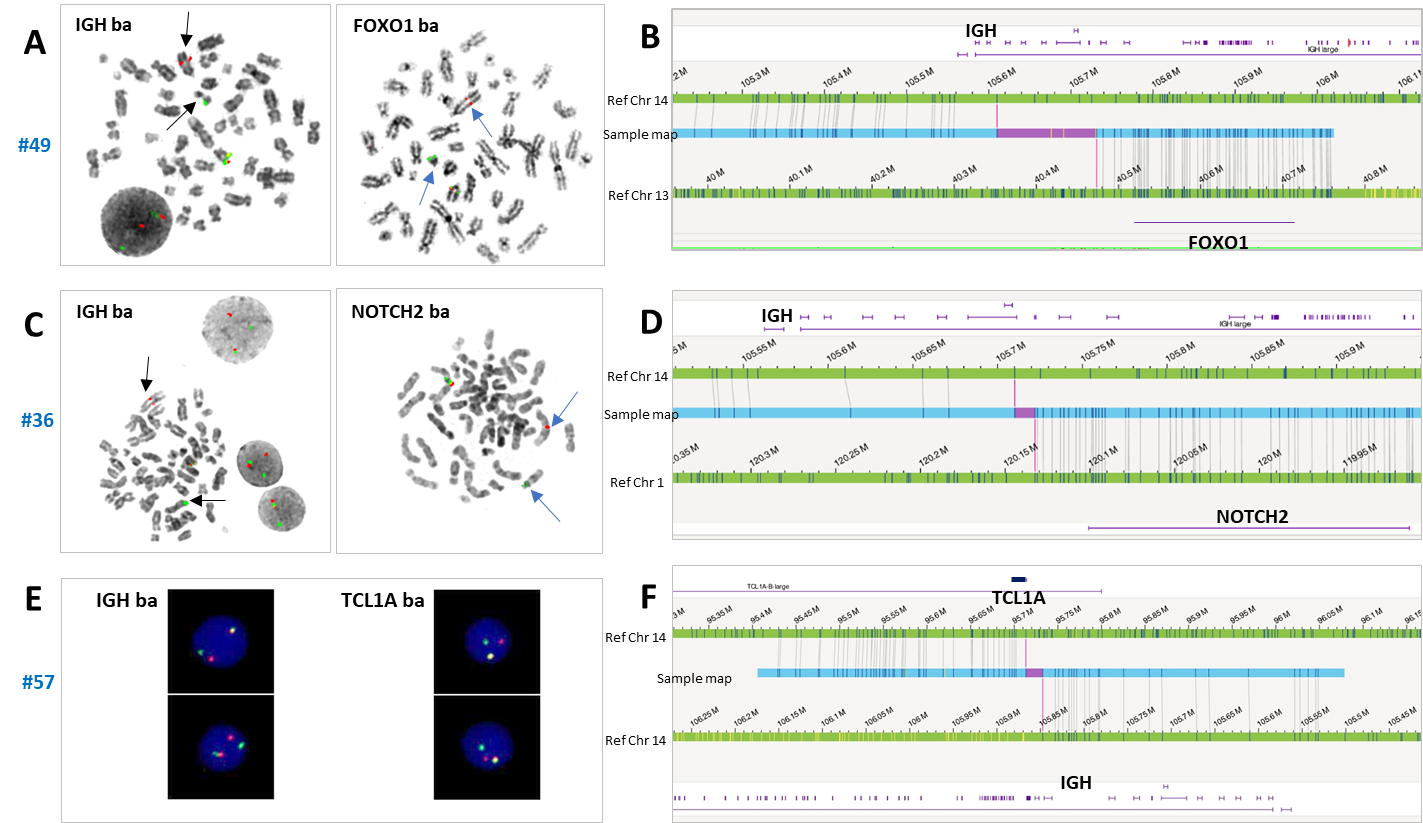
**

**Supplemental Figure 4. Identification of rare or novel IG rearrangements of undetermined significance.**

(A,B) Patient #49, Nodal marginal zone lymphoma. (A) Metaphase FISH showing splitting of the IGH ba probe (black arrows) and of the FOXO1 ba probe (blue arrows) due to t(13;14) translocation. (B) OGM genome browser view showing the t(13;14)/IGH::*FOXO1* rearrangement. (C,D) Patient #36, diffuse large B-cell lymphoma with t(1;14;3)(p12;q32;q27). (C) Metaphase FISH showing splitting of the IGH ba probe (black arrows) and of the NOTCH2 ba probe (blue arrows). (D) Genome browser view of OGM showing the t(1;14)/IGH::*NOTCH2* rearrangement. (E,F) Patient #57, lymphoplasmacytic lymphoma with a normal karyotype. (E) Interphase FISH showing splitting of both the IGH ba and TCL1A ba probes. (F) OGM genome browser view showing the IGH::*TCL1A* rearrangement. The name of each probe is indicated to the left of the corresponding FISH image. ba, breakapart.

**
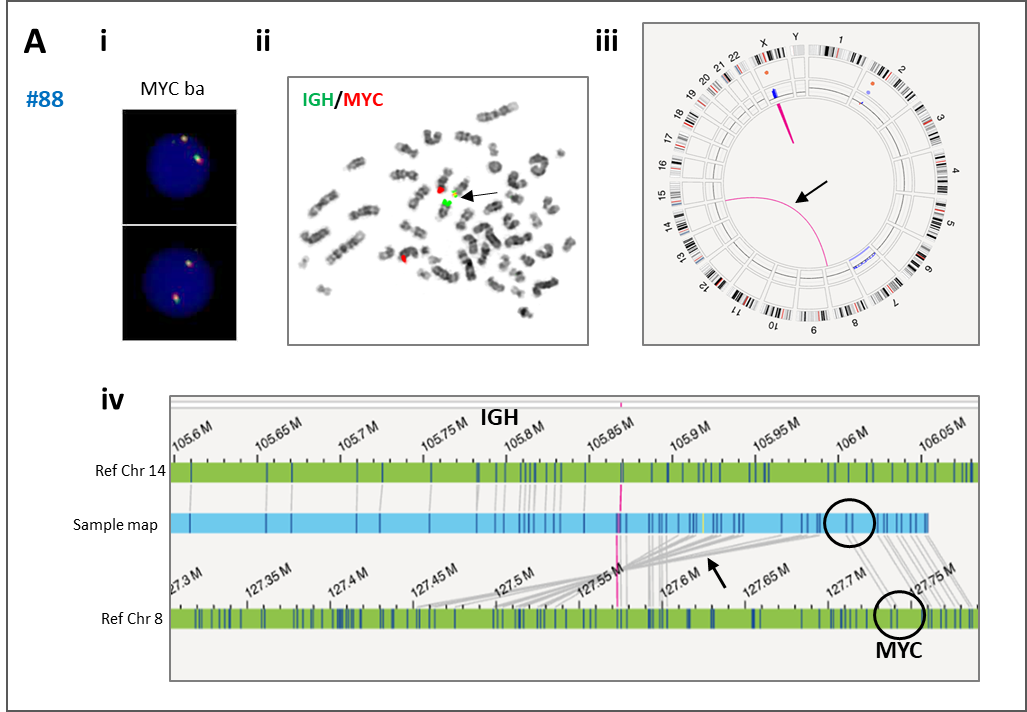
**


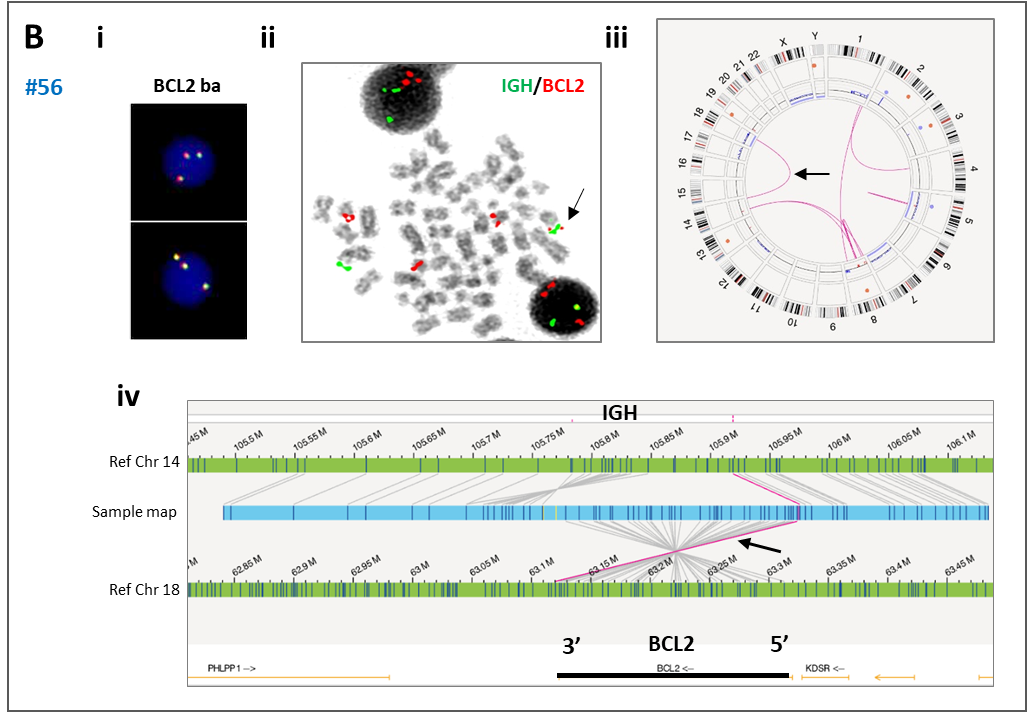


**
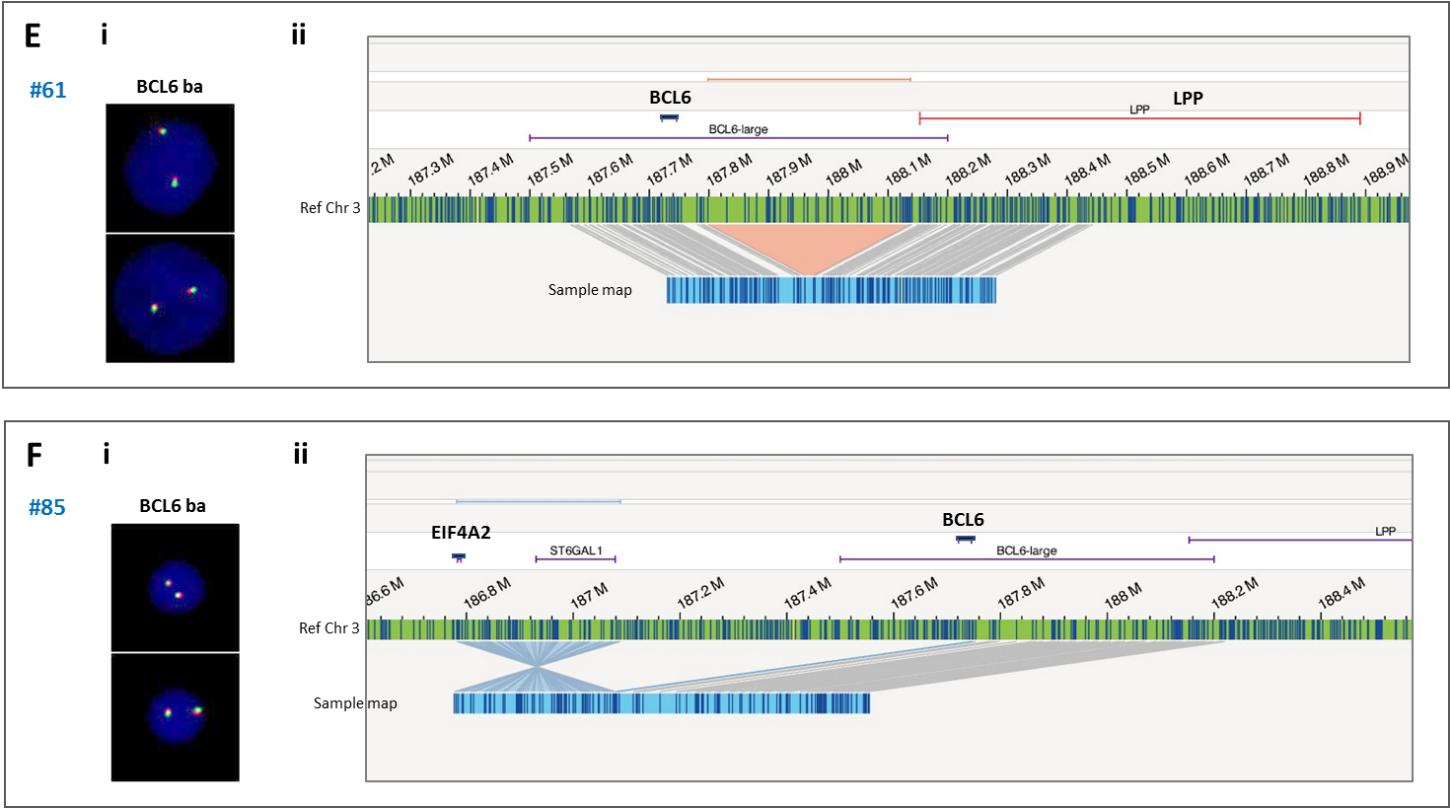

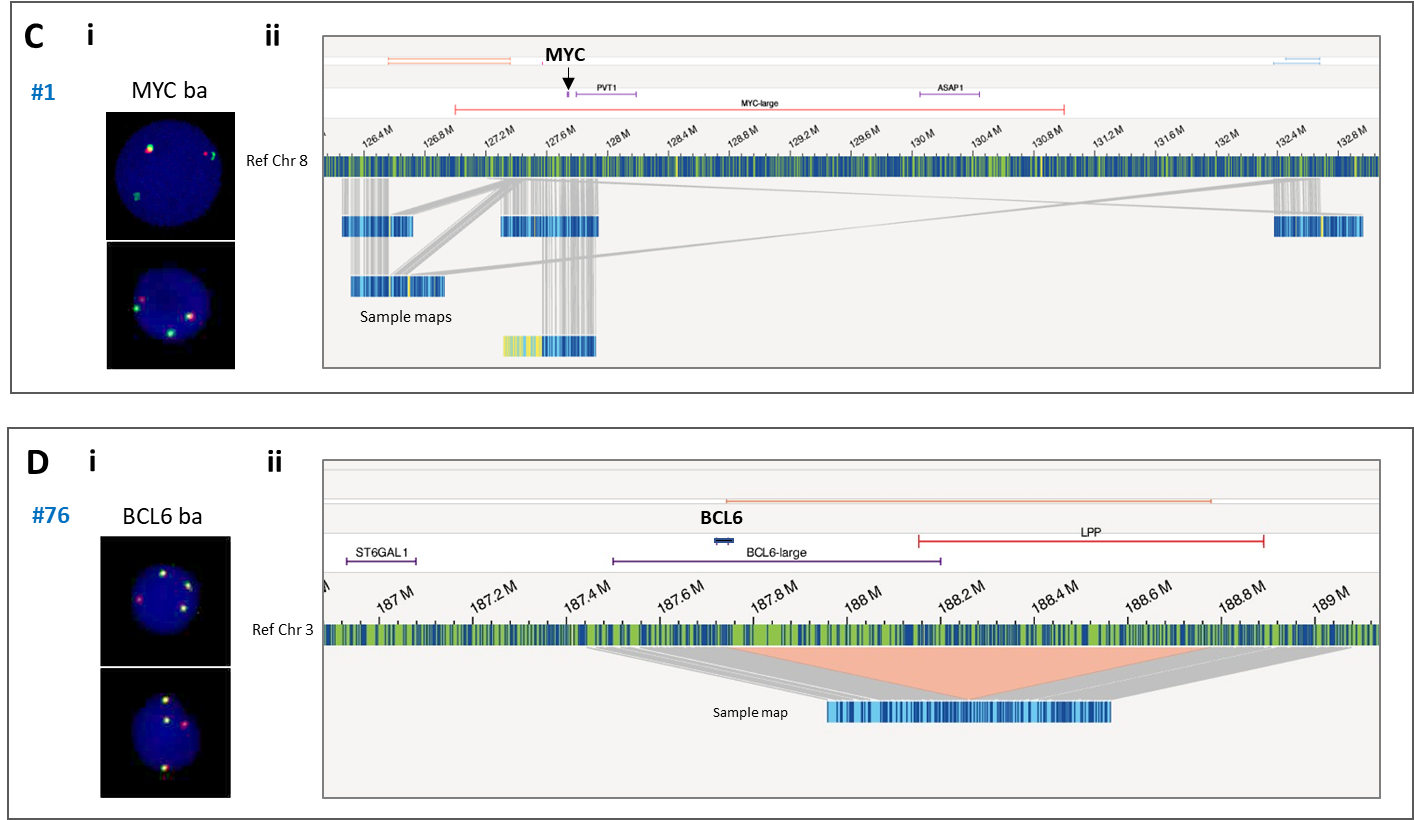
**

**Supplemental Figure 5. OGM detected diagnostically cryptic rearrangements.**

(A) Patient #88, Burkitt lymphoma with *MYC* insertion into the IGH locus. (i) Interphase FISH showing a normal pattern using the MYC ba probe. (ii) Metaphase FISH using a dual fusion IGH/MYC probe showing a thin fusion signal (black arrow), indicating insertion of *MYC* into the IGH locus. (iii) OGM Circos plot showing the IGH::*MYC* rearrangement (black arrow). (iv) Genome browser view showing the IGH::*MYC* rearrangement with an inverted 8q24.21 segment (black arrow) upstream of the *MYC* gene (black circle).

(B) Patient #56, follicular lymphoma with *BCL2* insertion into the IGH locus. (i) Interphase FISH using a BCL2 ba probe showing three fusion signals (consistent with trisomy 18). (ii) Metaphase FISH using a dual fusion IGH/BCL2 probe showing a fusion signal (black arrow). (iii) OGM Circos plot showing the t(14;18)/IGH::*BCL2* rearrangement. (iv) Genome browser view revealing an insertion with inversion (black arrow) of the *BCL2* gene in the IGH locus.

(C) Patient #1, diffuse large B-cell lymphoma with complex *MYC* rearrangement. (i) Interphase FISH using a MYC ba probe showing an “unbalanced gain” pattern (two fusion signals, one green signal). (ii) OGM genome browser view of the 8q24.21 region displaying a complex pattern around the *MYC* gene with numerous inversions.

(D) Patient #76, diffuse large B-cell lymphoma with tetraploid karyotype and *BCL6* rearrangement. (i) Interphase FISH using a BCL6 ba probe showing an “unbalanced gain” pattern (three fusion signals, one green signal). (ii) OGM genome browser view showing the deleted segment (5’ to BCL6 and into the LPP gene) indicating an *LPP::BCL6* rearrangement.

(E) Patient #61, high-grade B-cell lymphoma with *MYC* and *BCL6* rearrangements. (i) Interphase FISH with the BCL6 ba probe showing a normal pattern. (ii) Deleted segment boundaries localize to 5′BCL6 and 5′LPP, indicating a *LPP::BCL6* rearrangement.

(F) Patient #85, follicular lymphoma with *BCL6* rearrangement. (i) Interphase FISH with the BCL6 ba probe showing a normal pattern. (ii) OGM genome browser view showing an inverted segment localized to 5’*BCL6* and *EIF4A2*, indicating an *EIF4A2::BCL6* rearrangement.

The name of each probe is indicated at the top of the corresponding FISH image. ba, breakapart.

**
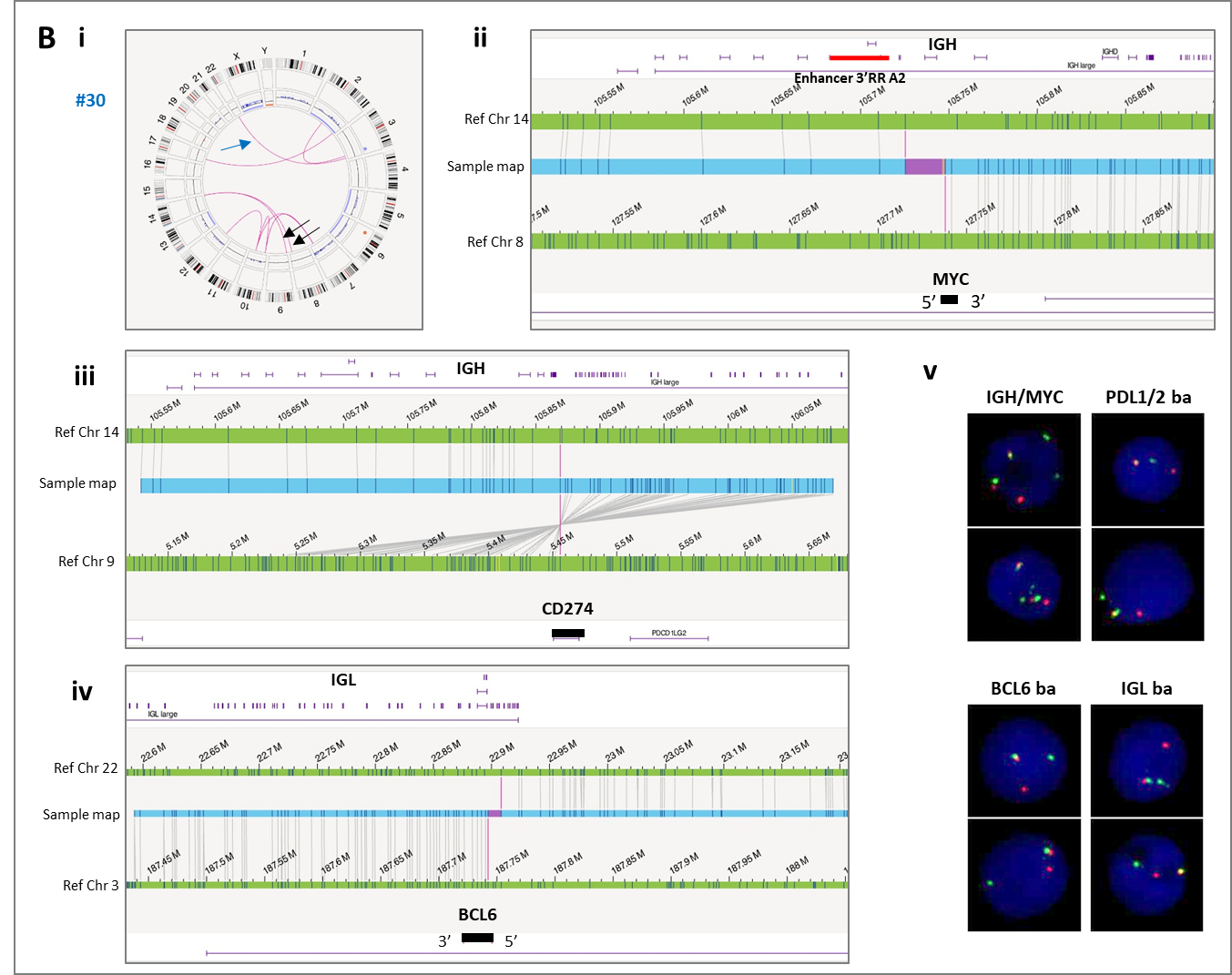

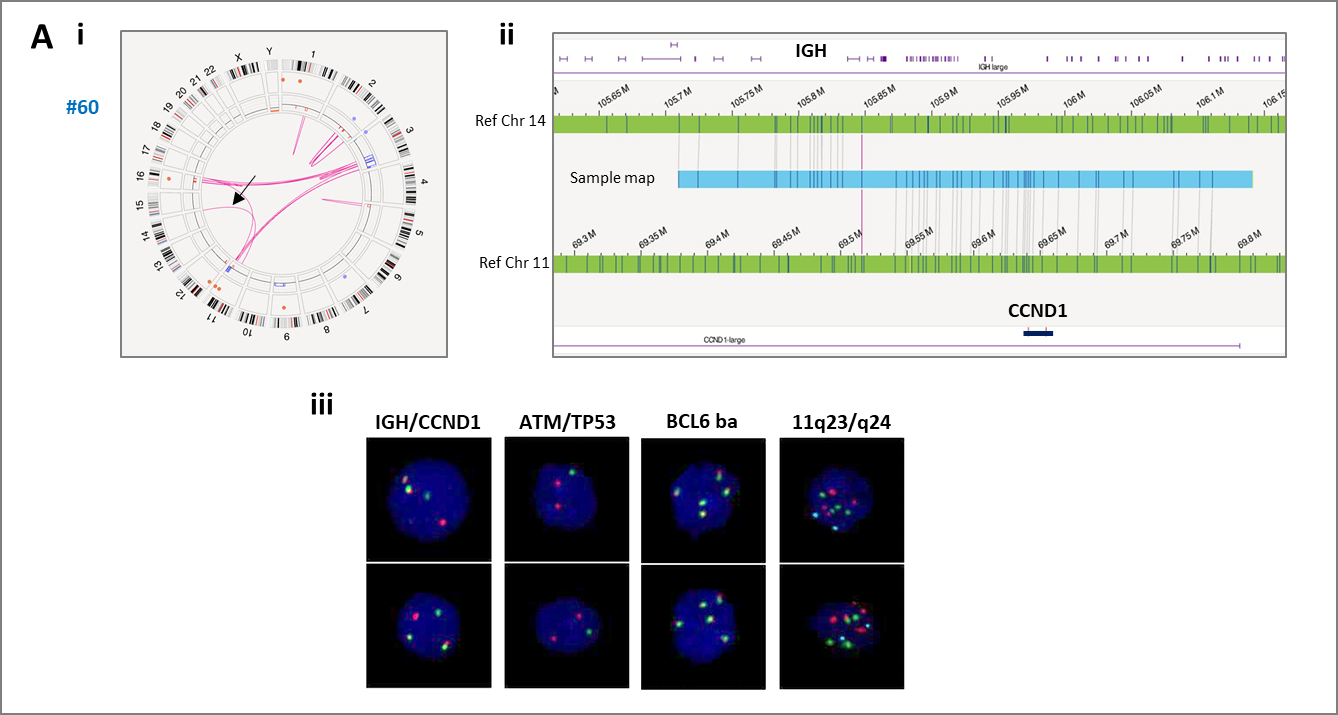
**

**Supplemental Figure 6. Diagnostic relevance of OGM in cases of non-informative karyotype.**

(A) Patient #60, mantle cell lymphoma with isolated loss of chromosome Y. (i) Circos plot showing a t(11;14)/IGH::*CCCND1* translocation (black arrow) and a chromoanagenesis pattern impacting chromosomes 3, 11, and 16. (ii) OGM genome browser view showing the IGH::*CCND1* rearrangement. (iii) Confirmation by interphase FISH: double fusion signals using the IGH/*CCND1* dual fusion probe; *ATM* deletion (loss of green signal); *BCL6* gain (five fusion signals); and 11q23q24 gain (four red and four green signals). (B) Patient #30, high-grade B-cell lymphoma with *MYC* and *BCL6* rearrangements and unsuccessful karyotype. (i) Circos plot showing dual IGH rearrangements involving *MYC* and *CD274/PDCD1LG2* (black arrows), and IGL::*BCL6* rearrangement (blue arrow). (ii) Genomic characterisation of the IGH::*MYC* rearrangement, showing a breakpoint near the IGH 3’RR A2 Enhancer (horizontal red line) and a 5′*MYC* breakpoint. (iii) The t(9;14)/IGH::*CD274/PDCD1LG2* rearrangement suggests a breakpoint within the *CD274* gene. (iv) IGL::*BCL6* rearrangement with 5’BCL6 breakpoint. (v) Confirmation by interphase FISH, showing a dual fusion profile for IGH::*MYC* rearrangement (the additional small green signal corresponds to the second IGH rearrangement). Split signal patterns are observed for *PDL1/PDL2*, *BCL6*, and IGL, using breakapart probes. The name of each probe is indicated at the top of the corresponding FISH image. ba, breakapart.

**
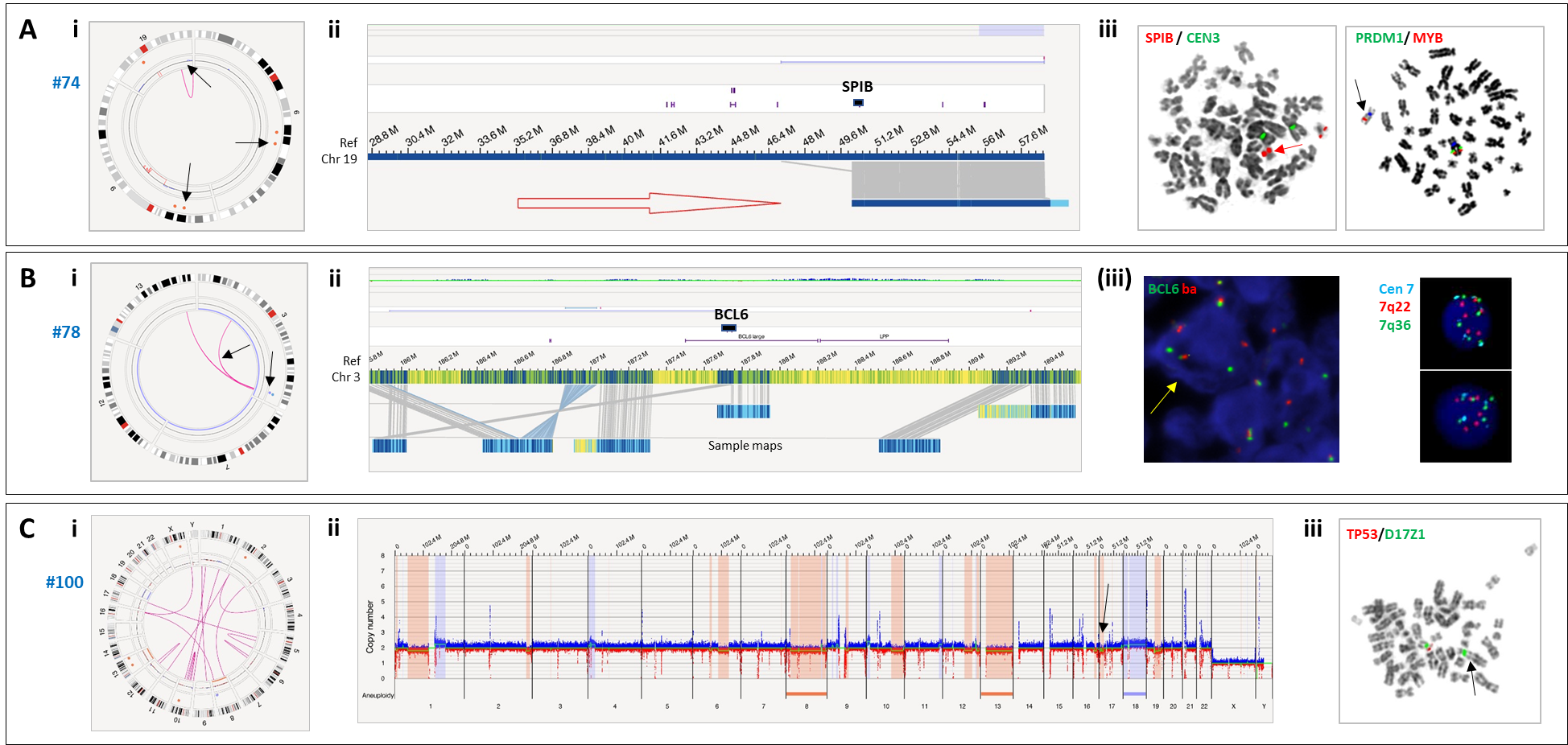
**

**Supplemental Figure 7**

**Illustration of OGM and FISH results for the three patients with initial uncertain diagnosis.**

(A) Patient #74, diffuse large B-cell lymphoma diagnosed through combined OGM and NGS. (i) Circos plot of chromosomes 6, 9 and 19 showing both *CDKN2A* and *PRDM1* losses, and *SPIB* gain (black arrows). (ii) Genome browser view depicting the gain of 11 Mb at 19q13, encompassing *SPIB*. (iii) FISH confirmation of *SPIB* gain (red arrow, red high-intensity signal) and *PRDM1* loss (black arrow, no green signal) . (B) Patient #78, transformation of marginal zone lymphoma diagnosed by integrating OGM, immunophenotyping, and clonality data. (i) Circos plot of chromosomes 3, 7, 12 and 13, showing multiple SVs involving 3q27 and 13q33 regions (black arrows). (ii) Genome browser view of the 3q27 region showing multiple sample maps (in blue) with a complex *BCL6* rearrangement. (iii) Interphase FISH confirmation showing splitting of the BCL6 ba probe (yellow arrow) and gain of chromosome 7 (tricolor probe Cen7/7q22/7q36) (six blue, red and green signals). (C) Patient #100, peripheral T-cell lymphoma, not otherwise specified, diagnosed based on OGM results, immunophenotyping, and T-cell clonality. (i) Circos plot revealing a complex genomic profile with numerous SVs and CNVs. (ii) Whole genome view showing multiple aneusomies and CNVs, including a 17p deletion (black arrow). (iii) FISH confirmation showing a *TP53* deletion: loss of red signal on derivative der(17) (black arrow).
